# Hypnogram and Hypnodensity Analysis of REM Sleep Behaviour Disorder Using Both EEG and HRV‐Based Sleep Staging Models

**DOI:** 10.1111/jsr.70046

**Published:** 2025-03-12

**Authors:** Jaap F. van der Aar, Merel M. van Gilst, Daan A. van den Ende, Hans van Gorp, Peter Anderer, Angelique Pijpers, Pedro Fonseca, Elisabetta Peri, Sebastiaan Overeem

**Affiliations:** ^1^ Department of Electrical Engineering Eindhoven University of Technology Eindhoven the Netherlands; ^2^ Philips Sleep and Respiratory Care Eindhoven the Netherlands; ^3^ Center for Sleep Medicine Kempenhaeghe Heeze the Netherlands; ^4^ Philips Innovation & Strategy Department of Innovation Engineering Eindhoven the Netherlands; ^5^ The Siesta Group Schlafanalyse Gmbh Vienna Austria

**Keywords:** electroencephalography, hypnodensity, obstructive sleep apnea, photoplethysmography, REM sleep behaviour disorder, sleep structure

## Abstract

Rapid‐eye‐movement (REM) sleep behaviour disorder (RBD) is a primary sleep disorder strongly associated with Parkinson's disease. Assessing sleep structure in RBD is important for understanding the underlying pathophysiology and developing diagnostic methods. However, the performance of automated sleep stage classification (ASSC) models is considered suboptimal in RBD, for both models utilising neurological signals (“ExG”: EEG, EOG, and chin EMG) and heart rate variability combined with body movements (HRVm). Here, we explore this underperformance through the categorical representation of sleep macrostructure (i.e., hypnogram) and a representation that leverages the underlying probability distribution of ASSCs (i.e., hypnodensity). By comparing the RBD population (*n* = 36) to a sex‐ and age‐matched group of OSA patients chosen for their anticipated similarly decreased sleep stability, we confirm lower 4‐stage classification performance in both ExG‐based ASSC (RBD: κ = 0.74, OSA: κ = 0.80) and HRVm‐based ASSC (RBD: κ = 0.50, OSA: κ = 0.63). Stages showing lower agreement in RBD, namely, N1 + N2 and REM sleep, exhibited elevated ambiguity in the hypnodensity, indicating more ambiguous classification distributions. Limited differences in bout durations between RBD and OSA suggested sleep instability is not necessarily driving lower agreement in RBD. However, stage transitions in OSA showed more abrupt changes in the underlying probability distribution, while RBD transitions had a more continuous profile, possibly complicating classification. Although both ExG‐based and HRVm‐based automated sleep staging in RBD remain challenging, hypnodensity analysis is informative for the characterisation of (RBD) sleep and can capture potential drivers of classification disagreement.

## 1 Introduction

Rapid‐eye‐movement (REM) sleep behaviour disorder (RBD) is a parasomnia characterised by dream‐enacting behaviour through movement and vocalisation caused by the loss of the normal muscle atonia during REM sleep (Boeve et al. 2007). In the absence of other associated medical conditions during diagnosis, RBD is categorised as isolated RBD (iRBD) (Sateia 2014). In recent years, iRBD has been recognised as a prodromal marker for α‐synucleinopathies: long‐term follow‐up studies show that most RBD patients develop Parkinson's Disease (PD), with a conversion rate above 90% (Galbiati et al. 2019; Iranzo et al. 2014; Schenck et al. 2013). Hence, interest in monitoring sleep in RBD is growing, but achieving reliable sleep measures appears more challenging than in other populations.

Conventionally, sleep is assessed with single‐night polysomnography (PSG). Information from the electroencephalography (EEG), chin electromyography (EMG), and electrooculography (EOG) is employed to manually assign one of the five stages (Wake/N1/N2/N3/REM) to each 30‐s epoch (Troester et al. 2023). Given the time‐consuming nature of manual scoring, many automated sleep stage classification (ASSC) models have been developed, mainly utilising neurological activity. Such models, hereafter referred to as “ExG‐based ASSC,” can reach comparable or even superior inter‐rater reliability compared with manual scorers (Bakker et al. 2023; Fiorillo et al. 2019). Indicators of sleep extend beyond neurological activity, encompassing various physiological processes, including the autonomic nervous system (Trinder et al. 2001). Hence, a second common group of ASSC models utilises the expression of sympathetic and parasympathetic activation in heart rate variability (HRV), often combined with measures of body movement derived from accelerometry to distinguish between wake and sleep. We will refer to these models as HRV and movement‐based, or HRVm‐based ASSC. Although these models may demonstrate lower sleep staging performance than their ExG‐based counterparts (Fonseca et al. 2023; Korkalainen et al. 2020a; Wulterkens et al. 2021), they can fulfil a distinct purpose: by leveraging less obtrusive sensors such as patches and wrist‐worn devices (Sartor et al. 2018; Zavanelli et al. 2021), they enable the measurement of sleep for extended periods of time.

The expression of sleep in cortical and autonomic processes may be impaired in RBD due to the (early) presence of neurodegeneration and autonomic dysfunction linked to α‐synucleinopathy. ExG‐based ASSC models, including combined EEG, EOG, and chin EMG, combined EEG and EOG, single‐channel EEG, and single‐channel EOG, all reported decreased performance in RBD as compared to healthy sleepers, to patients with insomnia, OSA, and to heterogeneous sleep‐disordered populations (Andreotti et al. 2018; Cesari et al. 2024; Cooray et al. 2019; Cooray et al. 2021; van der Aar et al. 2024). Similarly, in HRVm‐based ASSC models, the lowest agreement across sleep disorders is observed in a REM parasomnia cohort which primarily included RBD patients (Fonseca et al. 2020; Wulterkens et al. 2021). Furthermore, ASSC has revealed increased (REM) sleep instability in RBD, with more stage transitions and shorter REM bout durations (i.e., consecutive time spent in a sleep stage), potentially complicating classification performance (Christensen et al. 2016).

In general, we enforce categorical decisions upon the ASSC models by requiring the assignment of a single sleep stage to each epoch based on the class with the highest probability. This mirrors the process in manual scoring and produces a discrete hypnogram. The current body of work on RBD sleep staging performance and sleep stability in RBD relies on the analysis of these hypnograms. However, ASSCs offer novel opportunities for the representation of sleep structure. A currently mostly unexploited benefit of ASSCs is their ability to utilise the underlying sleep stage probability distributions provided by the model, referred to as the hypnodensity (Stephansen et al. 2018). Hypnodensity distributions may provide a more nuanced view of sleep structure compared to the categorical decisions. Furthermore, hypnodensities represent inter‐rater variability observed in manual scoring, enabling the exploration of ambiguity in sleep scoring without requiring multiple manual scorers (Bakker et al. 2023; Huijben et al. 2023; Stephansen et al. 2018). Hypnodensity‐derived ambiguity quantification has previously been used to estimate classification uncertainty (Mikkelsen et al. 2020; Phan et al. 2022), and may help understand why elevated disagreement between manual scoring and ASSC occurs in RBD. In addition, hypnodensity analysis can quantify the epoch‐to‐epoch change in the underlying probability distribution during stage transitions (i.e., transition continuity), allowing characterisation of previously observed decreased sleep stability in RBD (Christensen et al. 2016). Moreover, a recent study has shown that applying image recognition on hypnodensities can be used to discriminate between patients with RBD and controls, suggesting the hypnodensities of patients with RBD may hold clinically relevant information (Feuerstein et al. 2024).

In this study, we aim to first confirm earlier findings on decreased sleep staging performance in both ExG‐based and HRVm‐based ASSC models. Next, we study whether decreased sleep stability is observed in the RBD population using the discrete representation of sleep stages (i.e., the hypnograms). Last, and most importantly, we use a probability‐based approach by deriving ambiguity and continuity measures from the hypnodensities. We explore whether such measures can improve our understanding of RBD sleep staging and of the observed sleep macrostructure in RBD when using automated models that leverage different physiological processes.

## 2 Methods

### 2.1 Data

We sampled data from the PSG recordings of the Sleep and Obstructive Sleep Apnea Measuring with Non‐Invasive Applications (SOMNIA) database recorded before January 2021 (van Gilst et al. 2019). SOMNIA data were acquired at the Kempenhaeghe Center for Sleep Medicine (Heeze, the Netherlands) among individuals scheduled for an overnight PSG as part of the standard clinical routine. Each PSG recording was manually annotated by a single scorer, out of a pool of trained technicians, in accordance with AASM standards (Berry et al. 2017). Guidelines active in 2021 were used for the scoring of REM sleep in subjects with (suspected) RBD. Manual scoring was converted to a 4‐stage classification for the current study by combining N1 and N2 (Wake/N1 + N2/N3/REM). Sleep disorder diagnoses were coded according to the criteria specified in the International Classification of Sleep Disorders version 3 (ICSD‐3) (Sateia 2014). Guidelines active in 2021 were used for REM scoring in subjects with (suspected) RBD, but no major differences with the most recent guidelines are expected (Cesari et al. 2022).

We assembled two groups, an RBD group, and a mild‐to‐moderate OSA control group. The OSA control group was selected because of the presence of disturbed sleep and similar increased sleep fragmentation, but where fragmentation is not necessarily attributed to impaired sleep regulation (Bianchi et al. 2010; Korkalainen et al. 2020b; Mannarino et al. 2012). Moreover, the selected population allowed us to sample a control group from the same database using age matching since ageing can negatively impact sleep staging performance (Wulterkens et al. 2021). For the RBD group, we first included all available subjects with RBD as the primary diagnosis. From the available RBD patients, we excluded two subjects with comorbid severe OSA, defined by an apnea‐hypopnea (AHI) above 30. Severe OSA patients were excluded because of the expected and observed excessive levels of sleep fragmentation. For 20 out of the 36 RBD subjects, the RBD was isolated, while in 16 subjects the RBD was secondary due to the presence of parkinsonism. For the OSA control group, only patients with mild‐to‐moderate OSA (5 < AHI < 30) and without any other known sleep comorbidities were selected. Positive airway pressure (PAP) devices were not used during the PSG. Age matching was performed by randomly selecting an OSA subject for each RBD subject, with an age difference ≤ 5 years for each match.

### 2.2 ExG‐Based ASSC

Somnolyzer is a supervised deep learning ASSC model that classifies 30‐s epochs as either Wake, N1, N2, N3 or REM sleep (Anderer et al. 2022). All available frontal, central and occipital EEG channels (F4‐M1, F3‐M2, C4‐M1, C3‐M2, O2‐M1, O1‐M2), left and right EOG channels (E1‐M2, E2‐M2), and one chin EMG derivation (Chin1–ChinZ) were used for input (further referred to as ExG‐based ASSC), which were acquired at 512 Hz. Filtering was performed according to the AASM standards for EEG (0.3–35 Hz), EOG (0.3–35 Hz), and EMG (0.3–100 Hz), including a 50 Hz notch filter. The Somnolyzer model has been validated in 426 PSGs across multiple sleep‐disordered datasets, showing a 0.74 Cohen's Kappa agreement coefficient (κ) (Cohen 1960) versus manual scoring for the 5‐stage comparison (Anderer et al. 2022). Furthermore, analysis in six, nine and twelve manual scorers has revealed higher agreement between Somnolyzer and manual scoring than between individual manual scorers and their consensus vote (Bakker et al. 2023). Recently, Somnolyzer was approved by the AASM autoscoring certification program (https://aasm.org/about/industry‐programs/autoscoring‐certification/). Notably, due to the hierarchical structure of Somnolyzer, it is possible for a stage to be classified despite not having the highest probability. We refer to the original work for more information (Anderer et al. 2022). For the present study, after performing classification with Somnolyzer on the complete dataset, the N1 and N2 stages, as well as their hypnodensity‐derived probabilities, were combined in a single N1 + N2 stage to obtain a 4‐stage classification (Wake/N1 + N2/N3/REM) to allow direct comparison with the HRVm‐based ASSC.

### 2.3 HRVm‐Based ASSC

The present study made use of a previously trained and described neural network developed for 4‐stage (W/N1 + N2/N3/REM) sleep classification based on cardiac activity and body movements (further referred to as HRVm‐based sleep staging) (Fonseca et al. 2023). For this study, input signals were derived from a wrist‐worn watch‐like device (Sartor et al. 2018) containing reflective photoplethysmography using two green LED sources (32 Hz) and triaxial accelerometry (128 Hz) sensors, an earlier prototype of the Philips Healthband (Royal Philips, Amsterdam, The Netherlands). For the current study, we have applied the previously trained model as is, without any retraining (Fonseca et al. 2023). This model has been trained on PSGs with concurrent PPG and accelerometry recordings from various datasets (van Gilst et al. 2019; Klosh et al. 2001; van Meulen et al. 2023; Punjabi et al. 2015) containing both healthy subjects and sleep‐disordered patients (*n* = 1113). This model has shown a κ = 0.64 versus manual scoring in a hold‐out validation set (*n* = 394) containing also a heterogeneous pool of healthy subjects and sleep‐disordered patients (Fonseca et al. 2023), higher than most reported HRVm‐based ASSC models, which typically have been validated exclusively in healthy sleepers.

### 2.4 REM Atonia Index

The REM atonia index (RAI) was implemented for the automated detection of REM sleep without atonia (RSWA), resulting in one RAI value per subject which indicates reduced (RAI < 0.8), ambiguous (0.8 < RAI < 0.9), and non‐substantial (RAI > 0.9) loss of atonia (Cesari et al. 2018; Ferri et al. 2010). The Chin1‐ChinZ EMG derivation was used, substituted by the Chin2‐ChinZ in case of large, visually observed artefacts. No further artefact removal was applied. Signals were bandpass filtered between 10 and 100 Hz, including a 50 Hz Notch filter, sampled at 512 Hz.

### 2.5 Outcome Measures

To measure the performance of the ASSC models, we analysed the agreement between manual scoring and ExG‐based ASSC, as well as between manual scoring and HRVm‐based ASSC. *Overall ASSC performance* was calculated using the Kappa statistic, a multi‐class classification metric that accounts for the possibility of correct classification by chance (Cohen 1960). Besides overall ASSC performance for 4‐stage classification, additional 5‐stage performance for the ExG‐based ASSC is performed to allow comparison with published kappa values. S*leep stage‐specific ASSC performance* was calculated using the per‐class F1‐scores to simultaneously capture the sensitivity and positive predictive value (PPV) of the model.

*Sleep stability* was measured using bout duration, defined as the consecutive time spent in a sleep stage without a transition to another sleep stage, as indicated by each of the sleep‐scoring methods.

*Ambiguity* in the hypodensity was calculated using a normalised version of the Shannon entropy (Shannon 1948), which measures the spread of a probability distribution using the following formula:

$$\text{Ambiguity}=-\frac{1}{\log\left(4\right)}\;\sum_{i}p\left(x_{i}\right)\log p\left(x_{i}\right)$$

where *i* corresponds to each sleep stage class, and *p*(*x*_*i*_) to the probability of sleep stage *i* for a given epoch. By dividing Shannon entropy by *log*(*4*), since we perform 4‐stage classification, the entropy is normalised to a value between 0 and 1. When all but one sleep stage have a probability of 0, the entropy of that epoch will be 0. When all sleep stages have an equal probability, that epoch will have an entropy of 1. Entropy > 0.5 is only obtained when a non‐zero probability is given to at least three sleep stages, while entropy > 0.79 is only achieved when a non‐zero probability is given to all four sleep stages.

*Transition continuity* in the hypnodensity of ExG‐based and HRVm‐based ASSC was calculated when the hypnogram of the corresponding automated model indicated a sleep stage transition. Transition continuity was defined as the change in the probability distribution between the epoch before and the epoch after the stage transition, using the following formula:

Transition continuity=1−12∑ipxit−pxit+111

where *p*(*x*_*i*_) resembles the probabilities given to each sleep stage in a certain epoch. The formula calculates the sum of the absolute differences (i.e., for all sleep stages) in probability values between two adjacent epochs which represent a sleep stage transition. By multiplying the outcome by half and deducting it from 1, continuity is normalised to a value between 0 and 1. When there is maximum change in the probability distribution between two adjacent epochs during a stage transition, the continuity is 0, while continuity is 1 when there is no change.

### 2.6 Statistical Comparisons

For each analysis of the outcome measures, comparisons between the RBD and OSA groups within each scoring method were made, followed by a comparison between scoring methods. To correct for multiple testing, a Benjamini‐Hochberg correction was applied, allowing for a false discovery rate of 10% (Benjamini and Hochberg 1995). Adjusted *p* values were calculated by multiplying the *p* value by the total number of tests and dividing by its rank, correcting for any non‐decreasing order (Yekutieli and Benjamini 1999). *T*‐tests were performed to study differences in Kappa between ExG‐based and HRVm‐based overall ASSC performance, as well as between RBD and OSA groups. For all other outcome measures, due to the non‐normal distribution of data (Shapiro–Wilk test, not further reported), non‐parametric tests were performed, and the rank‐biserial correlation was reported as effect size (Kerby 2014). For sleep stability, a Friedman test was performed in advance to compare the three scoring methods.

## 3 Results

### 3.1 Clinical and Demographic Data

We assessed sleep recordings of 36 RBD and 36 age‐ and sex‐matched OSA subjects. An overview of the clinical and demographic data, as well as the sleep statistics derived from manual scoring, can be found in Table 1. As expected for AHI, significant differences between groups were observed, although AHI was also mildly elevated in the RBD group (RBD: 9.7; OSA: 19.2). For the RBD group, the most common sleep disorder comorbidities included mild‐to‐moderate OSA (*n* = 13; 5 > AHI > 30, excluding subjects with AHI > 30), insomnia (*n* = 6), and periodic limb movement disorder (*n* = 3). Hypersomnia, restless legs syndrome, delayed sleep–wake phase disorder, and sleep‐related laryngospasm were present in one subject each.

**TABLE 1:** Demographic information and sleep statistics derived from manual scoring of the 36 RBD and 36 OSA subjects, including statistical differences between the groups denoted by an asterisk when significant after adjusting *p* values for multiple testing. No tests were performed on age and sex since groups were matched on these characteristics. Means and standard deviations are reported, except for AHI, where medians and interquartile ranges are shown.

	RBD (*n* = 36)	OSA (*n* = 36)	Statistical comparison
Age (yrs.)	66.0 ± 6.5	64.0 ± 6.8	
Sex (m/f)	26/10	26/10	
AHI	9.7 (12.3)	19.2 (13.3)	*U* = 379, *p* = 0.002, **adj‐*p***_**(1)**_ = 0.02*
BMI	25.9 ± 3.6	26.8 ± 4.1	*T* = 0.87, *p* = 0.39, adj‐*p*_(5)_ = 0.54
Time in Bed (min.)	497 ± 38	508 ± 47	*T* = 1.01, *p* = 0.32, adj‐*p*_(4)_ = 0.54
Stage W (%)	23.5 ± 12.8	20.3 ± 11.1	*T* = 1.14, *p* = 0.26, adj‐*p*_(2)_ = 0.54
Stage N1 + N2 (%)	51.2 ± 11.0	53.8 ± 9.3	*T* = 1.11, *p* = 0.27, adj‐*p*_(3)_ = 0.54
Stage N3 (%)	12.8 ± 6.9	13.8 ± 6.8	*T* = 0.63, *p* = 0.53, adj‐*p*_(6)_ = 0.62
Stage REM (%)	12.5 ± 5.6	12.1 ± 4.6	*T* = 0.38, *p* = 0.70, adj‐*p*_(7)_ = 0.70

Abbreviations: AHI, Apnea‐hypopnea index; BMI, Body Mass Index.

### 3.2 ASSC Performance

For ExG‐based 4‐stage ASSC, in comparison with the OSA group, the RBD group presented a significantly lower Kappa (κ = 0.74 ± 0.11 versus κ = 0.80 ± 0.08 for OSA) and significantly lower median F1‐scores for N1 + N2 and REM sleep classification. For HRVm‐based 4‐stage ASSC, a similar pattern was observed: in comparison with the OSA group, RBD presented a significantly lower Kappa score (κ = 0.50 ± 0.16 versus κ = 0.63 ± 0.14 for OSA) and significantly lower median F1‐scores for N1 + N2, N3, and REM classification. Lower agreement can be observed especially in REM classification for RBD as compared to OSA, decreasing by nearly 20%–30% in all comparisons. In RBD, manually scored REM sleep was more frequently classified by the ASSCs as N1 + N2 sleep, leading to lower REM sensitivity and lower N1 + N2 PPV.

Detailed statistical analyses on differences between RBD and OSA in ASSC performance can be found in Table S1 of the Supporting Information, including 5‐stage overall performance, and 4‐stage overall and stage‐specific performance. Furthermore, Figure S2 illustrates 4‐stage classification confusion matrices.

### 3.3 Sleep Stability

**FIGURE 1:**
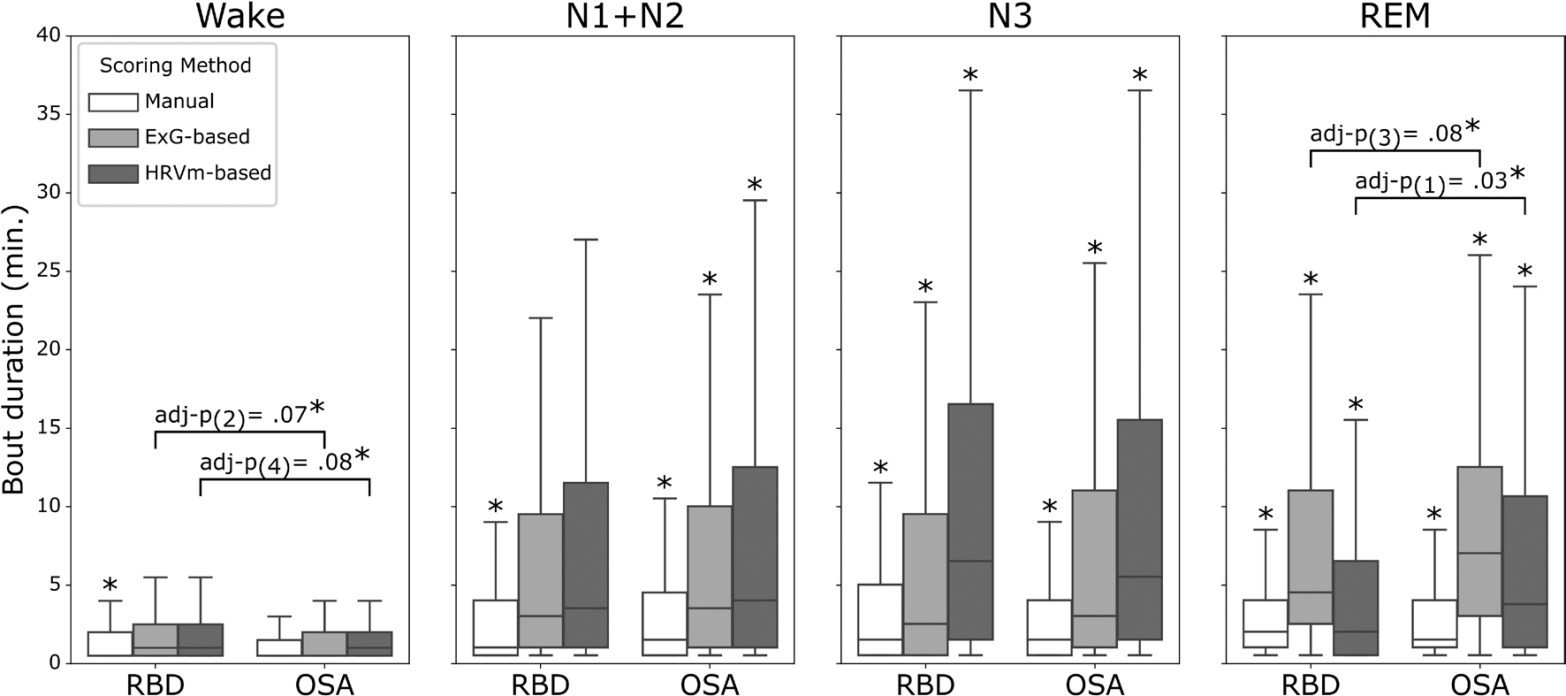
Distribution of bout durations for each of the sleep staging methods, visualised per sleep stage for RBD and OSA. Sleep staging methods included manual scoring (white), ExG‐based ASSC (light grey), and HRVm‐based ASSC (dark grey). For differences between RBD and OSA within scoring methods, adjusted *p* values are shown when significant. For between‐method comparisons, differences are denoted by an asterisk when bout durations significantly differ (after adjustment) from the other two methods.

Figure 1 illustrates the distribution of all bout durations for each group and each scoring method in minutes per sleep stage, totalling 20,857 bouts. Wake bout durations were shorter for OSA in the ASSC methods, not in manual scoring. REM bout durations were shorter in RBD in ASSC methods, but again not in manual scoring. No significant differences were found for other bout duration comparisons between RBD and OSA. A detailed statistical comparison between RBD and OSA can be found in the Table S3.

To study differences in bout durations between scoring methods, the bout durations of all sleep stages and both populations within a scoring method were aggregated. Significant differences in bout durations were found among the three scoring methods (*H*(2) = 437.80, *p* < 0.001, η^2^ = 0.02). Post hoc analyses showed that bout durations were shorter in manual scoring (median: 1.0, interquartile range (IQR): 3.0) than in ExG‐based ASSC (median: 2.0, IQR: 6.5; *U* = 2.45e^7^, *p* < 0.001, *r* = 0.15) and in HRVm‐based ASSC (median: 2.0, IQR: 8.0; *U* = 2.07e^7^, *p* < 0.001, *r* = 0.18). Although both ASSC methods presented a median bout duration of two minutes, significantly shorter bouts were observed in ExG‐based ASSC compared to HRVm‐based ASSC (*U* = 1.54e^7^, *p* = 0.008, *r* = 0.03). Sleep stage‐specific analyses showed significant differences in all comparisons, except in wake, as illustrated in Figure 1. A detailed statistical comparison between scoring methods can be found in Table S4.

### 3.4 Ambiguity

Hypnodensity‐derived ambiguity scores, calculated as the normalised entropy of the classification probability distribution, were evaluated for a total of 114,902 epochs. Table 2 indicates the overall and sleep stage‐specific statistical comparison between RBD and OSA. For both ASSC models, higher overall ambiguity was observed in RBD when compared to OSA. In ExG‐based ASSC, sleep stage‐specific ambiguity was higher for RBD in all sleep stages (N1 + N2, N3, and REM) except wake. In HRVm‐based ASSC, ambiguity was higher for RBD in wake, N1 + N2, and REM, but lower in N3.

**TABLE 2:** Statistical differences between RBD and OSA in hypnodensity‐derived ambiguity for both ExG‐based ASSC (left) and HRVm‐based ASSC (right). Ambiguity scores of all subjects within a population were aggregated; median values and interquartile ranges are reported. Sleep stages were split based on the classification of the ExG‐based and HRVm‐based ASSC models, not by manual scoring. Significant differences after multiple testing adjustment are denoted by an asterisk.

Stage	Ambiguity in ExG‐based ASSC			Ambiguity in HRVm‐based ASSC		
	RBD	OSA	Statistical comparison	RBD	OSA	Statistical comparison
Overall (4‐classes)	0.144 (0.355)	0.100 (0.277)	*U* = 7.23e^8^, *p* < 0.001, **adj‐*p***_**(2)**_ < 0.001*, *r* = 0.10	0.239 (0.353)	0.216 (0.333)	*U* = 6.83e^8^, *p* < 0.001, **adj‐*p***_**(7)**_ < 0.001*, *r* = 0.04
Wake	0.078 (0.160)	0.078 (0.173)	*U* = 3.17e^7^, *p* = 0.97, adj‐*p*_(10)_ = 0.97, *r* = 0.00	0.142 (0.361)	0.219 (0.377)	*U* = 2.74e^7^, *p* < 0.001, **adj‐*p***_**(6)**_ < 0.001*, *r* = 0.14
N1 + N2	0.141 (0.353)	0.096 (0.276)	*U* = 2.03e^8^, *p* < 0.001, **adj‐*p***_**(3)**_ < 0.001*, *r* = 0.12	0.238 (0.325)	0.197 (0.309)	*U* = 1.97e^8^, *p* < 0.001, **adj‐*p***_**(5)**_ < 0.001*, *r* = 0.09
N3	0.344 (0.350)	0.277 (0.358)	*U* = 1.26e^7^, *p* < 0.001, **adj‐*p***_**(8)**_ < 0.001*, *r* = 0.10	0.226 (0.307)	0.261 (0.310)	*U* = 1.10e^7^, *p* < 0.001, **adj‐*p***_**(9)**_ < 0.001*, *r* = 0.04
REM	0.276 (0.441)	0.087 (0.285)	*U* = 1.30e^7^, *p* < 0.001, **adj‐*p***_**(01)**_ < 0.001*, *r* = 0.33	0.426 (0.358)	0.270 (0.385)	*U* = 1.23e^7^, *p* < 0.001, **adj‐*p***_**(4)**_ < 0.001*, *r* = 0.26

**FIGURE 2:**
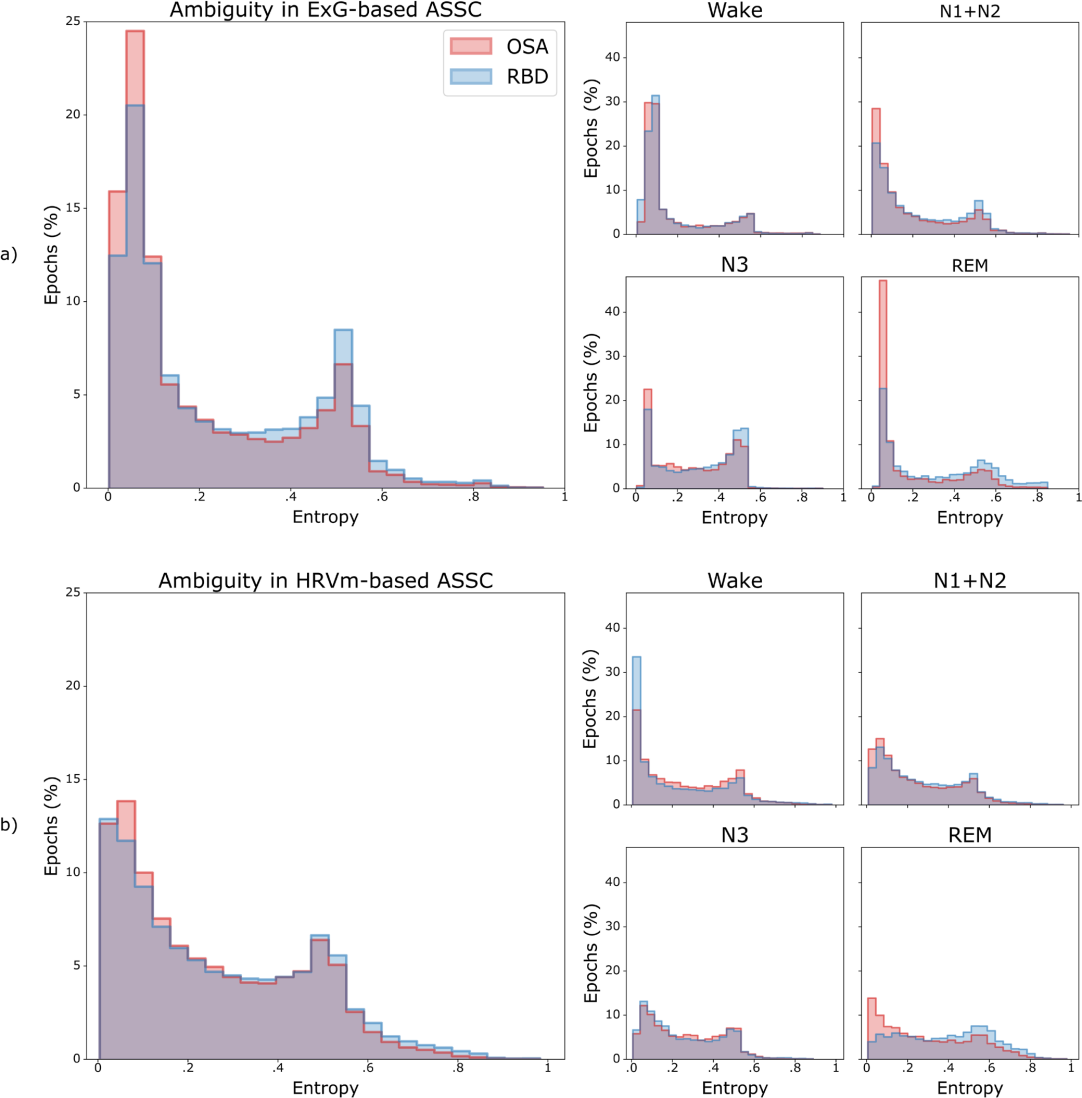
Distribution of hypnodensity‐derived ambiguity in ExG‐based ASSC (a) and HRVm‐based ASSC (b) for RBD (blue) and OSA (red) groups, with overlapping distribution in purple. Ambiguity distribution is shown for all epochs (left) and sleep stage‐specific (right), where the sleep stage corresponds with the sleep stage that the respective ASSC model classified. Y‐axis shows percentage of epochs (aggregated over all subjects within a population), x‐axis shows the ambiguity, measured as normalised entropy of the probability distribution in the hypnodensity.

Figure 2 illustrates the distribution of hypnodensity‐derived ambiguity in sleep stage classification for ExG‐based and HRVm‐based ASSC in both RBD and OSA, showing distinct distributions, especially for ambiguity in REM. Classification of REM sleep in RBD was accompanied by higher ambiguity in the probability distribution.

Regarding differences in overall ambiguity between the ASSC models, both in RBD and OSA, classification was less ambiguous in ExG‐based ASSC when compared to HRVm‐based ASSC (*U* = 5.30e^8^, *p* < 0.001, *r* = 0.17 for RBD; *U* = 5.02e^8^, *p* < 0.001, *r* = 0.25 for OSA).

### 3.5 Transition Continuity

**FIGURE 3:**
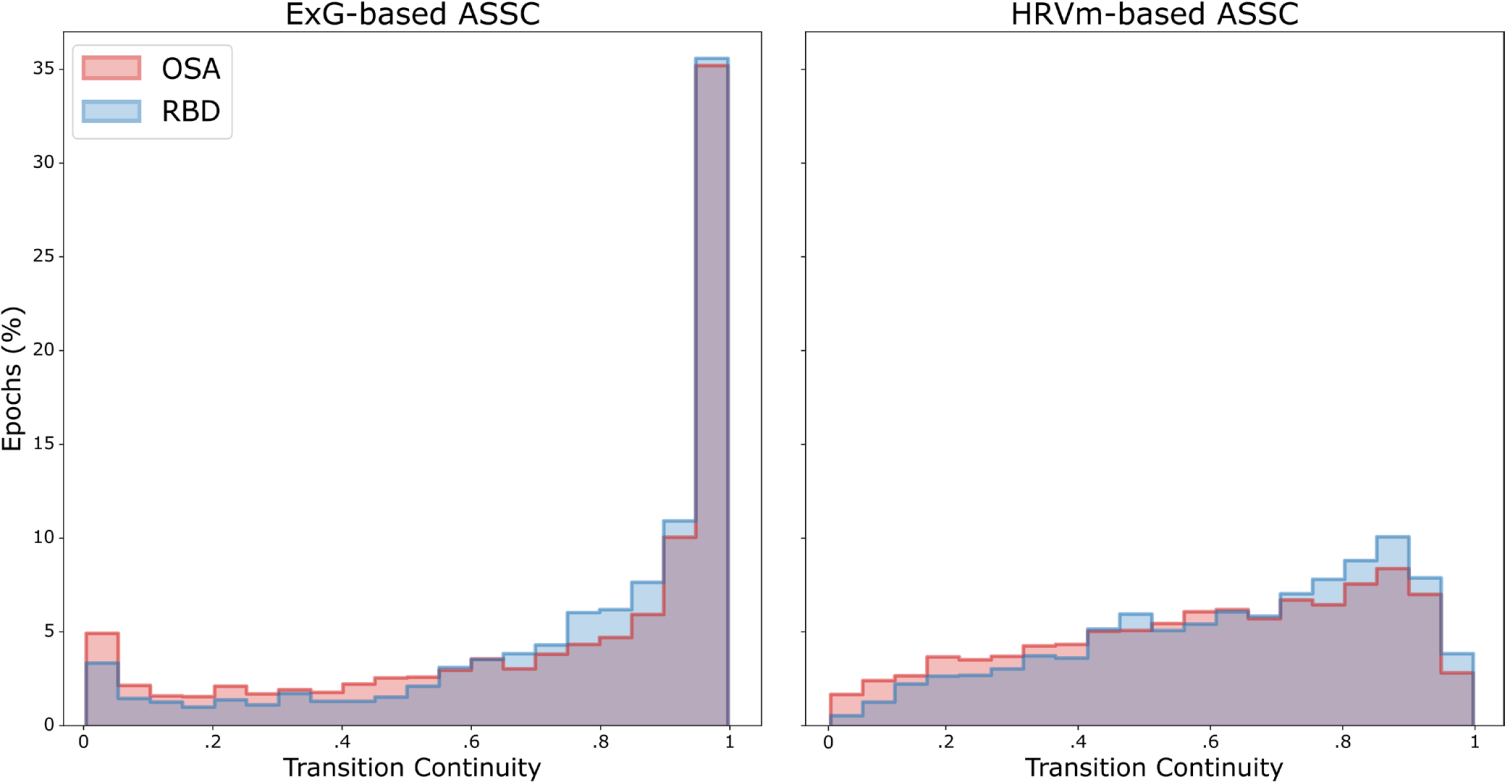
Distribution of sleep stage transition continuity in ExG‐based (left) and HRVm‐based sleep staging (right) for each transition epoch in the RBD (blue) and OSA (red) populations, with overlapping distribution in purple. Y‐axis shows percentage of epochs (aggregated over all subjects within a population), x‐axis shows the continuity score. Lower continuity indicates a larger change in the sleep stage probability distribution of the hypnodensity when the ASSC model indicates a sleep stage transition.

Figure 3 illustrates the transition continuity obtained using the ExG‐based and HRVm‐based models for both RBD and OSA. Continuity scores were evaluated for a total of 10,382 sleep stage transitions.

Sleep stage transitions in RBD (median: 0.888, IQR: 0.321) showed higher continuity when compared to OSA (median: 0.873, IQR: 0.430) for ExG‐based ASSC (*U* = 3.53e^6^, *p* = 0.002, *r* = 0.05). Similarly, for HRVm‐based ASSC, higher continuity was observed in RBD (median: 0.678, IQR: 0.370) than in OSA (median: 0.624, IQR: 0.405; *U* = 3.69e^6^, *p* < 0.001, *r* = 0.10). Hence, in OSA, sleep stage transitions showed larger changes in the probability distribution of the hypnodensity than in RBD.

Regarding differences in transition continuity between the ASSC models, higher continuity was observed in ExG‐based ASSC when compared to HRVm‐based ASSC in both RBD (*U* = 4.60e^6^, *p* < 0.001, *r* = 0.42) and in OSA (*U* = 4.80e^6^, *p* < 0.001, *r* = 0.38). Hence, ExG‐based ASSC transitions are associated with smaller changes in the probability distribution.

Notably, the ExG‐based ASSC shows a larger percentage of stage transitions with either very low or very high continuity compared to the HRVm‐based ASSC. When averaged over both populations, the ExG‐based ASSC exhibits roughly four times as many stage transitions with continuity below 0.05 (4.18% vs. 1.09%) and about 11 times as many stage transitions with continuity above 0.95 (36.07% vs. 3.36%). Hence, stage transitions in the ExG‐based ASSC are more often associated with either small changes (i.e., high continuity) or large changes (i.e., low continuity) in the probability distribution.

## 4 Discussion

This study aimed to characterise RBD sleep and improve understanding of RBD sleep staging by analysing the hypnograms and hypnodensities of two automated sleep stage classification (ASSC) models. These models leverage different physiological processes (cortical vs. autonomic) that are potentially impaired in individuals with RBD. We confirm earlier findings that the agreement of ASSC models with manual scoring is generally worse in RBD: for both ExG‐based and HRVm‐based ASSC, suboptimal performance was found in RBD when compared to age‐ and sex‐matched OSA patients. Limited differences between RBD and OSA were observed in bout durations, suggesting that sleep instability is not necessarily driving lower scoring agreement in RBD. However, stage transitions in OSA were associated with more abrupt changes in the probability distribution, while RBD transitions had a more continuous profile, which potentially complicates stage classification in RBD. Furthermore, sleep stages that showed lower performance in RBD, namely N1 + N2 and REM, exhibited higher ambiguity in the hypodensity, indicating more spread in probabilities during classification.

### 4.1 ASSC Performance

For ExG‐based ASSC, a large contributor to the lower sleep staging agreement in RBD was the misclassification of REM sleep as N1 + N2. Decreased REM sensitivity is well described in the literature (Andreotti et al. 2018; Cooray et al. 2019; van der Aar et al. 2024) and is, at least to some extent, attributed to REM sleep without atonia (RSWA), a distinct characteristic of RBD reflecting underlying pathophysiology (Boeve et al. 2007; Sateia 2014). The current study confirms these findings by reporting a positive relationship between the RAI and the agreement on REM classification (Figure S5). Interestingly, although the HRVm‐based ASSC model targets different physiological processes to measure sleep, similar patterns in terms of sleep stage‐specific performance were observed as with ExG‐based ASSC, again showing pronounced misclassification of REM sleep as N1 + N2. Possibly, this phenomenon can be attributed to RSWA as well. While the co‐occurrence of sympathetic activations and body movements can be present during non‐REM sleep (e.g., during arousals) (Tobaldini et al. 2013), in RBD, the simultaneous presence may also manifest during REM sleep due to the lack of muscle atonia, confusing the classifier. Notably, the chin EMG‐derived RAI did not correlate with agreement on REM classification for HRVm‐based ASSC (Figure S5), suggesting a different expression of RSWA in the wrist‐worn PPG and actigraphy acquired signals, but this should be further studied. Second, since REM sleep is characterised by an alternating pattern of sympathetic and parasympathetic activity (Tobaldini et al. 2013), it is possible that the presence of autonomic dysfunction as an early manifestation of α‐synucleinopathy in RBD blunts the sympathetic response in these patients, complicating REM classification. We refer to the Supporting Information for a more extensive interpretation of the ASSC performance results in RBD and the comparison to similar methods.

### 4.2 Sleep Stability

In iRBD, the underlying pathophysiological mechanisms affect the brainstem at an early stage, impacting the inhibitory loops that regulate sleep and wake, potentially causing sleep instability (Luppi et al. 2011). When comparing RBD bout durations with a matched control OSA group also characterised by increased instability (Bianchi et al. 2010; Korkalainen et al. 2020a; Mannarino et al. 2012), we found no or only small differences between groups. These results suggest that the number of transitions and consequently, the duration of uninterrupted sleep stage bouts are not necessarily driving the lower sleep staging performance we found in RBD. We only observed lower sleep stability in REM sleep of RBD patients using ExG‐based and HRVm‐based ASSC. This effect was not observed for manual scoring or in any of the other sleep stages. Interestingly, REM instability has been found previously in RBD when compared to healthy controls and patients with periodic limb movement disorder (PLMD), but also only in automated classification and not in manual scoring (Christensen et al. 2016), potentially because microstructural changes can be better captured by the ASSC models (Cesari et al. 2021). However, it is possible that the supposed REM instability is a mere byproduct of the ambiguity in the REM classification in RBD, which we further explored by analysing not the categorical classification of sleep stages (i.e., the hypnogram), but the underlying probability distribution (i.e., the hypnodensity).

**FIGURE 4:**
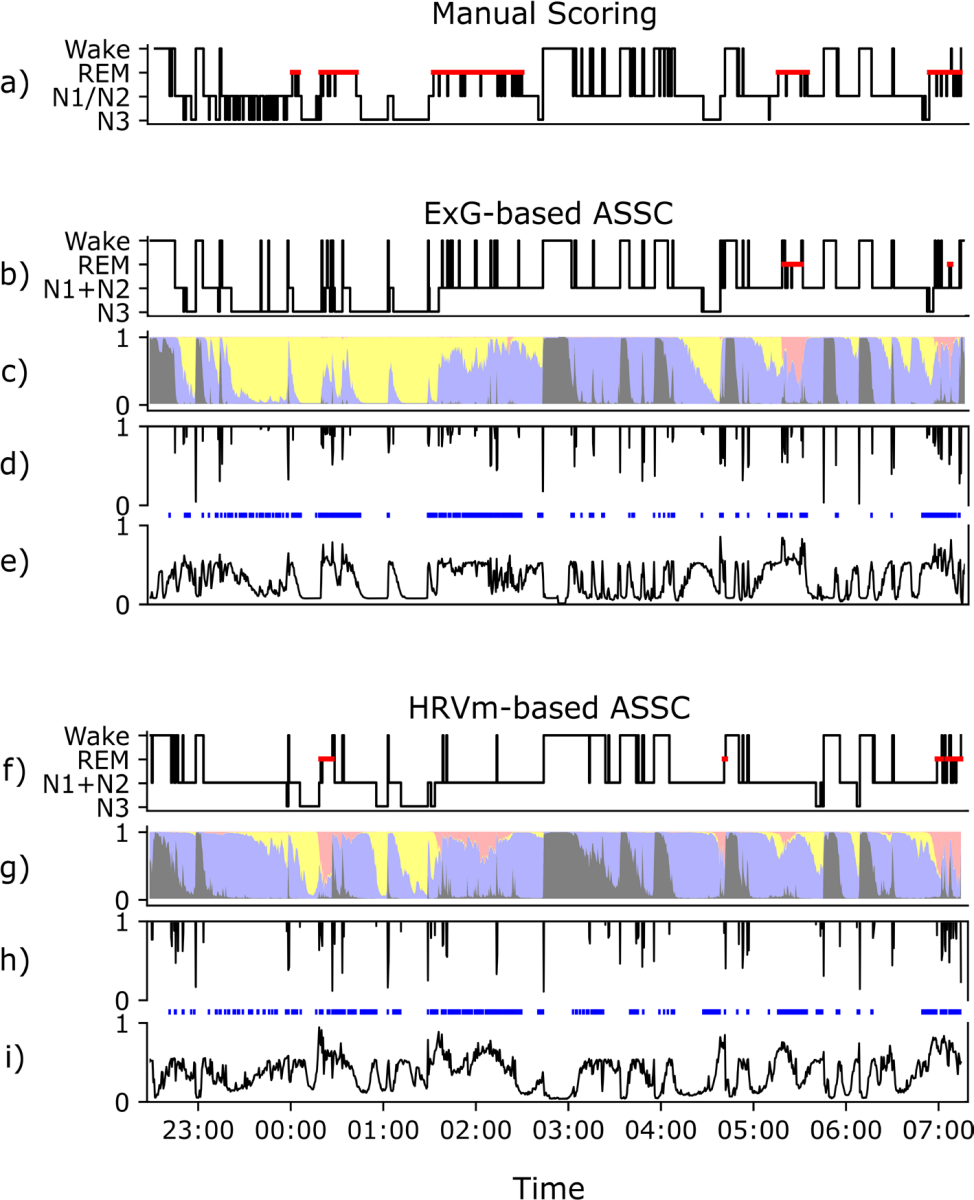
A representative example of sleep stage classification in a 66‐year‐old male patient with RBD and noticeable cognitive impairment, selected for the low REM classification agreement sleep in both automated ASSCs. Panel a shows the hypnogram obtained based on manual scoring. Panels b–e show the hypnogram (b), hypnodensity (c), transition continuity (d), and ambiguity (e) of ExG‐based ASSC; epochs with disagreement with manual scoring are indicated as blue ticks. Panels f–i show the same measures for HRVm‐based ASSC. The hypnodensities indicate the probability distributions of wake (dark grey), N1 + N2 (light blue‐magenta), N3 (yellow), and REM (light red) sleep. Multiple study findings can be observed in the figure. First, manually scored REM sleep was often missed by the ASSCs as N1 + N2, lowering the REM sensitivity and N1 + N2 PPV. For this subject, higher REM probabilities were observed in the HRVm‐based ASSC, but in general, higher REM probabilities were observed in the ExG‐based ASSC, as indicated by the higher agreement of the ExG‐based method with manual scoring. Second, most stage transitions are scored with manual scoring, and the least are transitions with HRVm‐based ASSC. Third, disagreement between manual scoring and the ASSCs is generally associated with higher ambiguity. Fourth, relatively less disagreement is observed in when transition continuity was low (i.e., longer continuity bars indicate lower continuity values) compared to transitions with high continuity. The misclassification in ExG‐based ASSC (panel b and c) during the first part of the night is likely not a feature of RBD but instead can be attributed to the mixing of pathological slow wave sleep during other sleep stages, including during REM sleep.

Furthermore, large differences in bout durations were observed between scoring methods, with the most transitions scored in manual and the fewest in HRVm‐based sleep staging. Comparing median bout duration values to existing research is complicated by differences in methodology and stage categorization, and by age‐ and disorder‐specific effects (Arnardóttir et al. 2010; Bianchi et al. 2010; Klerman et al. 2013; Wei et al. 2017). We hypothesise that the bout duration differences between manual scoring and automated ASSCs are mostly driven by short, non‐consensus intrusions of other sleep stages. While individual manual scorers may label them, automated models function as a consensus voting system that solely represents the majority class, resulting in a smoother hypnogram. This phenomenon can be observed in Figure 4 and in previous literature reporting inter‐rater agreement in relation to automated classification (Anderer et al. 2022; Bakker et al. 2023; van Gorp et al. 2023; Huijben et al. 2023). Moreover, the higher number of stage transitions scored in ExG‐based ASSC compared to HRVm‐based ASSC could be attributed to the ability of the modality to detect brief stage‐specific changes. For example, while short wake intrusions are relatively easily detected in EEG as arousals, they may not present as clearly in autonomic nervous system activity.

### 4.3 Hypnodensity‐Derived Characterisation

**FIGURE 5:**
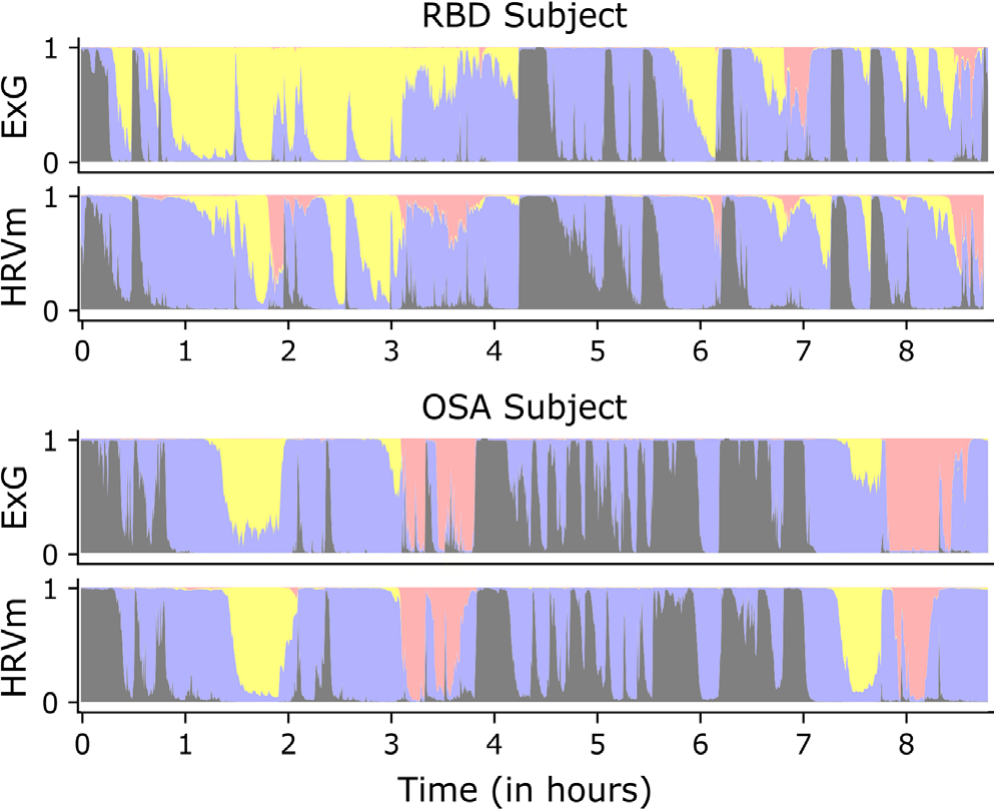
ExG‐based and HRVm‐based hypnodensities of a 66‐year‐old patient with RBD (same subject as illustrated in Figure 4) and an OSA subject with the same age. The hypnodensities indicate the probability distributions of wake (dark grey), N1 + N2 (light blue‐magenta), N3 (yellow), and REM (light red) sleep. In the RBD subject, REM probability typically failed to become the majority class in either ASSC, with probability assigned to multiple other stages concurrently (i.e., higher ambiguity). In contrast, REM classification in the OSA subject was less ambiguous. Furthermore, in general, RBD hypnodensities exhibited a more gradual transition of stage probabilities (i.e., higher continuity), whereas the OSA hypnodensities displayed abrupter changes, in both ASSCs. For example, in the OSA subject, the N3 and REM probabilities reached the majority class faster, and spikes of wake probability were sharper, when compared to the RBD subject.

This study did not only assess the previously used method of utilising neurological signals for hypnodensity analysis (Stephansen et al. 2018), but we extended this approach by applying it to sleep staging using HRVm. For both the ExG‐based and HRVm‐based ASSC models, we found higher ambiguity as characterised with hypnodensity in the sleep scoring of RBD when compared to OSA, with especially large differences and distinct distributions in REM sleep. When epochs were classified as REM sleep by one of the automated models, there was more ambiguity, indicating a larger spread in the probability distribution in the RBD population. In this group, more epochs showed an ambiguity score above 0.5, suggesting that a non‐zero sleep stage probability was assigned to at least three sleep stages in these epochs, usually REM, wake and N1 + N2. An illustrative example of this difference in REM sleep classification between RBD and OSA can be found in Figure 5. At least for the ExG‐based method, the REM ambiguity was associated with RSWA computed using the RAI detector (Figure S5), suggesting the ambiguous distributions may reflect the presence of RSWA. However, whether the ambiguous distributions are inherent characteristics of RBD sleep or a current limitation of the used models remains unclear. First, it is possible that the uncertain distributions arise because human raters would also score these epochs ambiguously, that is, there is elevated inter‐rater disagreement in these epochs (Bakker et al. 2023; Huijben et al. 2023). Second, ambiguous distributions may arise because the signals lack information representative of specific sleep stages. These two interpretations can be classified as aleatoric uncertainty. Third, increased ambiguity may represent uncertainty about the model's ability to identify stage‐specific characteristics because it lacked training examples representative of the observed patterns (epistemic uncertainty) (van Gorp et al. 2022). Epistemic uncertainty may be reduced by training a model specifically on a RBD population or on earlier sleep recordings of the individual, methods which both improve classification agreement, including the detection of REM sleep (van der Aar et al. 2024; Andreotti et al. 2018). Moreover, comparing the hypnodensities of automated sleep stagers to the inter‐rater variation in the manual scoring of RBD sleep recordings could infer additional information about the existence of epistemic and aleatoric uncertainty. In a previous study, it was shown that the ExG‐based ASSC model used here captured ambiguity comparable with the inter‐rater agreement of 6, 9 and 12 scorers in patients with sleep‐disordered breathing (Bakker et al. 2023). However, it is yet unknown whether this holds in an RBD population. Notably, since for Parkinson's Disease inter‐rater agreement is lowest across all tested (sleep) disorders (Bliwise et al. 2000; Danker‐Hopfe et al. 2004; Rosenberg and Van Hout 2013), we also expect lower inter‐rater agreement in RBD, which may contribute to the differences between manual and automated scoring. Future work should investigate the relationship between the captured ambiguity in ASSCs tuned to RBD characteristics and the inter‐rater agreement of a large number of human scorers in the RBD population.

Finally, we used the probability distributions to characterise sleep stage transitions in RBD and OSA. Although we observed small differences in the number of transitions between groups, the continuity of the transitions did show differences. In the OSA group, transitions were accompanied by lower continuity in the hypnodensities of the ASSC models, manifested as a larger shift in the probability distribution. Possibly, these more abrupt shifts in sleep architecture are related to (apnea‐induced) arousals prominently observed in OSA patients (Younes 2004), since higher AHI values were associated with lower continuity for the ExG‐based ASSC (Figure S6). Sample sizes were not large enough for analysis on specific sleep stage transitions, but we hypothesise these population differences are especially apparent in transitions affected by arousals, including transitions from N3 to wake or N1 + N2. In contrast, RBD transitions showed a more continuous hypnodensity profile. An illustrative example of the differences in continuity between RBD and OSA can be observed in Figure 5. These differences may partly explain the generally lower sleep staging performance in the RBD group: slower, less pronounced shifts in sleep stage‐specific patterns may lead to increased misclassification around transitions. As illustrated in Figure 4, larger shifts in the probability distribution (i.e., lower continuity) help establish clearly different patterns around transitions and lead to an overall increase in agreement between manual scoring and ASSC models. Such clear, unambiguous changes were four times more often present in ExG‐based ASSC compared to HRVm‐based ASSC when considering very low continuity (> 0.05). Furthermore, minimal shifts in the probability distribution (continuity > 0.95) were 11 times more often present in ExG‐based ASSC. Possibly, the ExG‐based ASSC can detect subtle changes in neurological signals that carry sleepstage‐pecific information, thus capturing more stage transitions, as discussed in the previous section. Figure 4 illustrates multiple instances in ExG‐based ASSC where probability around 0.5 is assigned to two stages; hence, a small change in the distribution causes a stage transition with high continuity.

### 4.4 Clinical Implications

Similar relative differences between RBD and OSA for (almost) all outcome measures were observed, including for overall and sleep stage‐specific performance, (REM) ambiguity, and continuity. However, both ASSC models show distinct distribution differences, suggesting the absolute values observed for the outcome measures are specific to the targeted modality and to how the model is trained. Therefore, these models are not interchangeable, and each serves a unique purpose. While the ExG‐based sensor set‐up will continue as standard for RBD diagnostics and clinical single‐night monitoring, the HRVm‐based method provides a less obtrusive solution for prolonged in‐home sleep monitoring. Since RBD often predates the clinical manifestation of α‐synucleinopathy by several years, sometimes even decades (Berg et al. 2015; Postuma et al. 2019), early detection of RBD is important and could offer a window for potential future neuroprotective interventions. Early stage RBD indications may be subtle (Figorilli et al. 2020), can exhibit night‐to‐night variability (Cygan et al. 2010), and include known risk factors (e.g., occupational pesticide exposure, head injury, and ageing (Postuma et al. 2012)). Hence, the population at risk for RBD and α‐synucleinopathy would be particularly suitable for in‐home screening, prolonged monitoring and follow‐up. When the ASSC models are employed, hypnodensity analyses can be used for further characterisation, including the use of ambiguity and continuity measures as indicators for scoring reliability, derived diagnostic markers, and potentially to distinguish between pathological and natural sleep (transitions) (Feuerstein et al. 2024). Moreover, while for both modalities automated sleep staging remains challenging, the reduced sleep staging performance, and in particular incorrect classification of REM sleep, in itself could potentially signal RBD.

### 4.5 Limitations

The RBD group can be considered a complicated patient population, consisting mostly of elderly individuals with sleep disorder comorbidities. Hence, with the limited sample size, not all heterogeneity in the population may be captured. Furthermore, almost all patients with RBD have developed or will eventually develop α‐synucleinopathy (Galbiati et al. 2019; Iranzo et al. 2014; Schenck et al. 2013). To study the influence of α‐synucleinopathy on the ASSC agreement and the hypnodensity‐derived metrics, subgroup analyses for isolated and secondary RBD were performed (Tables S7 and S8). However, no significant differences were observed, and these analyses were heavily restricted by further reduced sample sizes. While this study showed hypnodensity‐derived metrics can help characterise RBD sleep, further research should be conducted to study whether they contain diagnostic value. This should include larger RBD subgroups, manual (REM) sleep stage and RSWA scoring using the most recent guidelines (Cesari et al. 2022), and control groups with patients with differential diagnoses, including severe OSA, periodic limb movement disorder (PLMD), and other parasomnias (Iranzo and Santamaría 2005; Troester et al. 2023).

### 4.6 Conclusion

This study performed hypnogram and hypnodensity analysis on RBD using two automated sleep stage classification models that leveraged either neurological signals (“ExG”) or HRV and body movements. For both modalities, the discrete representation of sleep structure (i.e., the hypnogram) was inadequate in revealing complexities in RBD sleep staging when compared to OSA. In contrast, the underlying probability distribution (i.e., the hypnodensity) indicated more ambiguous classification and less distinct stage transitions in RBD. As such, hypnodensity‐derived measures have been shown to be informative for the characterisation of (RBD) sleep and allow studying potential drivers of classification disagreement.

## Author Contributions

**Jaap F. van der Aar:** conceptualization, methodology, investigation, formal analysis, writing – original draft, visualization. **Merel M. van Gilst:** conceptualization, methodology, supervision, writing – review and editing. **Daan A. van den Ende:** conceptualization, methodology, supervision, writing – review and editing. **Hans van Gorp:** conceptualization, methodology, writing – review and editing. **Peter Anderer:** conceptualization, software, writing – review and editing. **Angelique Pijpers:** writing – review and editing. **Pedro Fonseca:** conceptualization, methodology, supervision, writing – review and editing, software. **Elisabetta Peri:** conceptualization, methodology, supervision, writing – review and editing. **Sebastiaan Overeem:** conceptualization, methodology, supervision, writing – review and editing.

## Ethics Statement

This study utilised data from the SOMNIA database, recorded before January 2021. Data collection was reviewed and approved by the Maxima Medical Center medical ethical committee (Veldhoven, the Netherlands; N16.074). The data analysis protocol was approved by the medical ethical committee of the Kempenhaeghe Center for Sleep Medicine and the Philips Research Internal Committee for Biomedical Experiments. The study adhered to the guidelines of the Declaration of Helsinki, Good Clinical Practice, and current legal requirements. All patient data were de‐identified to protect participant privacy.

## Conflicts of Interest

At the time of writing, J.F.v.d.A., H.v.G., D.A.v.d.E., and P.F. were employed and/or affiliated with Royal Philips, a commercial company and manufacturer of consumer and medical electronic devices, commercialising products in the area of sleep diagnostics and sleep therapy. Philips had no role in the study design, decision to publish or preparation of the manuscript. S.O. received an unrestricted research grant from UCB Pharma and participated in advisory boards for UCB Pharma, Takeda, Jazz Pharmaceuticals and Bioprojet, all paid to the institution and all unrelated to the present work. P.A. is Chief Scientific Officer and shareholder of The Siesta Group, a service provider supporting the measurement of sleep, wakefulness and brain activity in clinical trials and research in the CNS field. The Siesta Group had no role in the study design, decision to publish or preparation of the manuscript. The other authors have no conflicts of interest.

## Supporting information


**Data S1.** Supporting Information.

## Data Availability

The SOMNIA data is available from the Center for Sleep Medicine Kempenhaeghe upon reasonable request (van Gilst et al. 2019). The data can be requested by presenting a scientific research question and by fulfilling all the regulations concerning the sharing of human data. The details of the agreement will depend on the purpose of the data request and the entity that is requesting the data (e.g., research institute or corporate). Each request will be evaluated by the Kempenhaeghe Research Board and, depending on the request, approval from independent medical ethical committees might be required. Access to data from outside the European Union will further depend on the expected duration of the activity; due to the work required from a regulatory point of view, the data is less suitable for activities that are time critical or require access on short notice. Specific restrictions apply to the availability of the data collected with sensors not comprised in the standard PSG set‐up, since these sensors are used under licence and are not publicly available. This data may however, be available from the authors with permission from the licensors. For inquiries regarding availability, please contact Merel van Gilst (m.m.v.gilst@tue.nl).
