# Supplementary material for: Hypnogram and Hypnodensity Analysis of REM Sleep Behaviour Disorder Using Both EEG and HRV‐Based Sleep Staging Models
Source: J Sleep Res. 2025 Mar 12;34(5):e70046. doi: 10.1111/jsr.70046 (PMC12426719; doi:10.1111/jsr.70046)
Supplement: Supplementary file 1 — Data S1. Supporting Information. [file JSR-34-e70046-s001.docx]

**ASSC Performance in RBD & OSA**

Table S1 illustrates the agreement between manual scoring and ExG-based ASSC, as well as between manual scoring and HRVm-based ASSC, for both RBD and OSA. For ExG-based 4-stage ASSC, in comparison with the OSA group, the RBD group presented a significantly lower Kappa (κ = .74 ± .11 versus κ = .80 ± .08 for OSA) and significantly lower median F1 scores for N1+N2 and REM sleep classification. For HRVm-based 4-stage ASSC, a similar pattern was observed: in comparison with the OSA group, RBD presented a significantly lower Kappa score (κ = .50 ± .16 versus κ = .63 ± .14 for OSA) and significantly lower median F1-scores for N1+N2, N3, and REM classification. To allow comparisons with published kappa values between ExG-based ASSC and manual scoring, we additionally performed a 5-stage comparison for the ExG-based ASSC, resulting in κ = .69 ± .10 for RBD and κ = .74 ± .08 for OSA.

Regarding the differences in overall 4-stage classification performance between sleep scoring methods, for RBD, significantly higher overall agreement was observed for ExG-based ASSC compared to HRVm-based ASSC (*T* = 7.49, *p* < .001, *d* = 1.76). For OSA, significantly higher agreement was also observed for ExG-based ASSC compared to HRVm-based ASSC (*T* = 6.71, *p* < .001, *d* = 1.58).

|  | **Agreement for ExG-based ASSC** | | |  | **Agreement for HRVm-based ASSC** | | |
| --- | --- | --- | --- | --- | --- | --- | --- |
| **Stage** | **RBD** | **OSA** | **Statistical Comparison** |  | **RBD** | **OSA** | **Statistical Comparison** |
| Overall  (5 classes) | κ = .69 ± .10 | κ = .74 ± .08 | *T* = 2.62, ***p* = .011**,  *d* = .62 |  | - | - | *-* |
| Overall  (4 classes) | κ = .74 ± .11 | κ = .80 ± .08 | *T*= 2.75, *p* = .008,  **adj-*p*_(5)_ = .02***,  d = .64 |  | κ = .50 ± .16 | κ = .63 ± .14 | *T* = 3.77, *p* < .001,  **adj-*p*_(3)_ = .001***,  *d* = .89 |
| Wake | F1 = 85.96 (14.60) | F1 = 87.53 (11.37) | *U* = 535, *p* = .21,  adj-*p*_(8)_ = .26,  *r* = .17 |  | F1 = 68.23 (18.95) | F1 = 73.43 (16.11) | *U* = 561, *p* = .33,  adj-*p*_(10)_ = .33,  *r* = .13 |
| N1+N2 | F1 = 87.03 (7.97) | F1 = 89.96  (3.54) | *U* = 375, *p* = .002,  **adj-*p*_(4)_ = .005***,  *r* = .42 |  | F1 = 73.85 (12.28) | F1 = 80.93 (10.34) | *U* = 329, *p* < .001,  **adj-*p*_(2)_ = .001***,  *r* = .49 |
| N3 | F1 = 78.35 (23.74) | F1 = 82.29 (15.24) | *U* = 552, *p* = .28,  adj-*p*_(9)_ = .31,  *r* = .15 |  | F1 = 54.74 (31.35) | F1 = 72.00 (28.32) | *U* = 465.5, *p* = .04,  **adj-*p*_(7)_ = .06***,  *r* = .28 |
| REM | F1 = 84.07 (13.39) | F1 = 89.58  (9.40) | *U* = 453, *p* = .03,  **adj-*p*_(6)_ = .05***,  *r* = .30 |  | F1 = 54.99 (36.02) | F1 = 76.89 (16.94) | *U* = 279.5, *p =* .001*,*  **adj-*p*_(1)_ < .001***,  *r* = .49 |

**Table S1.** Sleep stage classification agreement in RBD and OSA between (left) manual scoring and ExG-based ASSC (left), and between manual scoring and HRVm-based ASSC (right), for 5-class sleep staging (only for ExG-based ASSC), 4-class sleep staging, and per sleep stage for the 4-class classification. Overall classification performance indicates the mean and standard deviation per-subject Kappa agreement. Sleep stage-specific performance indicates the median per-subject F1-scores and interquartile ranges are shown. Significant differences are denoted by an asterisk when significant after controlling for false discovery.

**
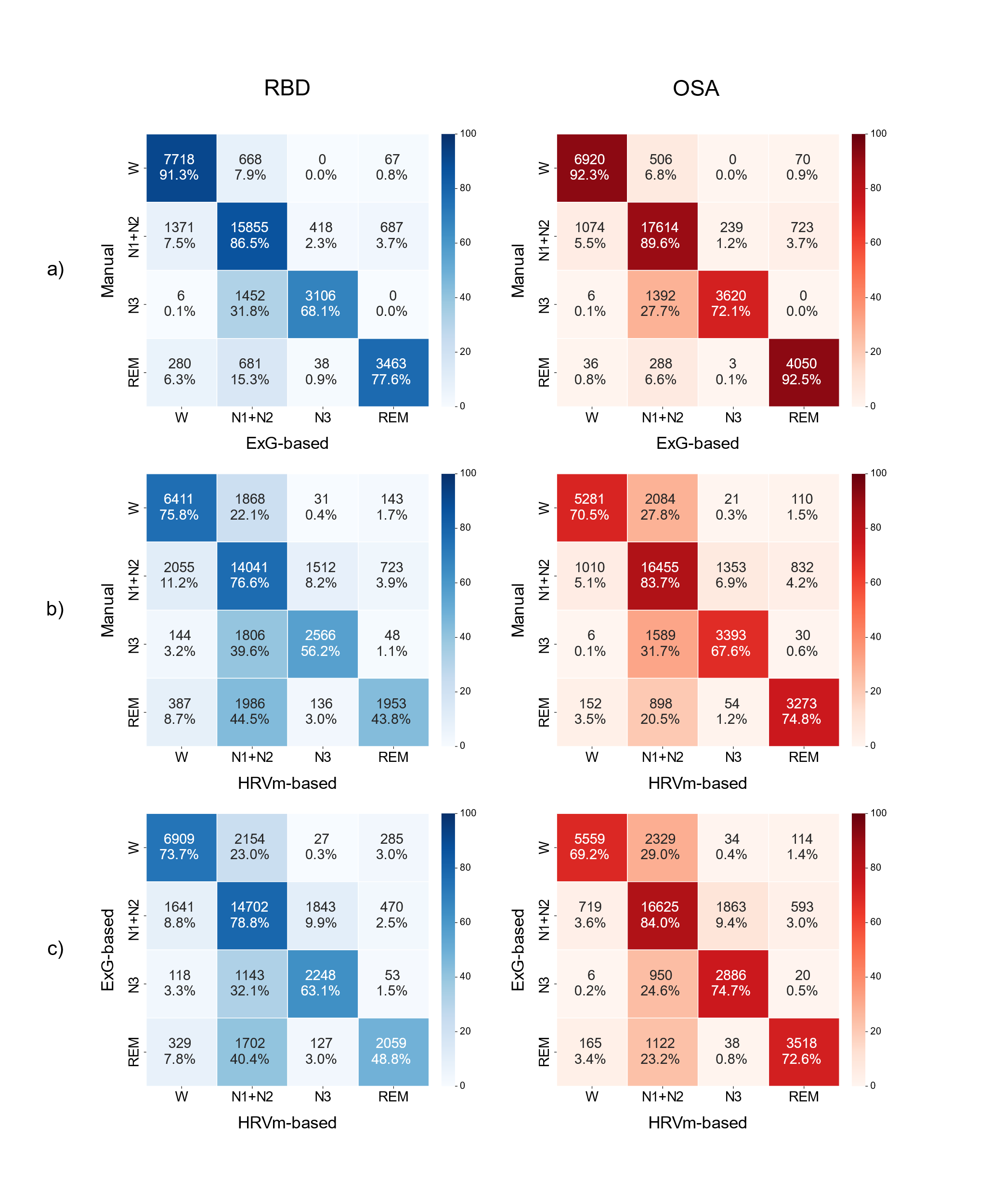
**Figure S2 illustrates 4-stage classification confusion matrices comparing manual scoring, ExG-based ASSC, and HRVm-based ASSC. The lower agreement in REM classification for RBD as compared to OSA is clearly visible, decreasing by nearly 20-30% in all comparisons. In RBD, manually scored REM sleep was more frequently classified as N1+N2 sleep, leading to lower REM sensitivity and lower N1+N2 PPV.

**Figure S2.** Confusion matrices between manual and ExG-based ASSC (a), manual and HRVm-based ASSC (b), and between ExG-based and HRVm-based ASSC (c), for the RBD (left, blue) and OSA (right, red) groups. Absolute number of epochs and percentage accuracy given. Darker color indicates higher accuracy.

**Sleep Stability**

All statistical differences in bout durations between populations and between scoring methods are shown in Table S3 and S4.

| **Stage** | **Scoring Method** | **RBD bout duration Median [IQR]** | **OSA bout duration Median [IQR]** | **Statistical comparison:  Mann-Whitney *U-*statistic, *p*-value, rank-biserial effect size, adjusted *p*-value** |
| --- | --- | --- | --- | --- |
| Wake | Manual | .5 [.5 – 2.0] | .5 [.5 – 1.5] | *U* = 9.11*e*^5^, *p* = .69,  *r* = -.008, adj-*p*_(11)_ = .70 |
|  | ExG-based | 1.0 [.5 – 2.5] | .5 [.5 – 2.0] | *U* = 6.81*e*^5^, *p* = .01,  *r* = -.06, **adj-*p*_(2)_ = .07*** |
|  | HRVm-based | 1.0 [.5 – 2.5] | .5 [.5 – 2.0] | *U* = 4.34*e*^5^, *p* = .03,  *r* = -.06, **adj-*p*_(4)_ = .08*** |
| N1+N2 | Manual | 1.0 [.5 – 4.0] | 1.5 [.5 – 4.5] | *U* = 2.49*e*^6^, *p* = .45,  *r* = .01, adj-*p*_(9)_ = .60 |
|  | ExG-based | 3.0 [1.0 – 9.5] | 3.5 [1.0 – 10.0] | *U* = 8.95*e*^5^, *p* = .25,  *r* = .03, adj-*p*_(7)_ = .41 |
|  | HRVm-based | 3.5 [1.0 – 11.5] | 4.0 [1.0 – 12.5] | *U* = 7.00*e*^5^, *p* = .09,  *r* = .04, adj-*p*_(5)_ = .21 |
| N3 | Manual | 1.5 [.5 – 5.0] | 1.5 [.5 – 4.0] | *U* = 1.53*e*^5^, *p* = .51,  *r* = -.02, adj-*p*_(10)_ = .61 |
|  | ExG-based | 2.5 [.5 – 9.5] | 3.0 [1.0 – 11.0] | *U* = 3.44*e*^4^, *p* = .27,  *r* = .05, adj-*p*_(8)_ = .41 |
|  | HRVm-based | 6.5 [1.5 – 16.5] | 5.5 [1.5 – 15.5] | *U* = 2.51*e*^4^, *p* = .70,  *r* = -.02, adj-*p*_(12)_ = .71 |
| REM | Manual | 2.0 [1.0 – 4.0] | 1.5 [1.0 – 4.0] | *U* = 2.11*e*^5^, *p* = .14,  *r* = -.05, adj-*p*_(6)_ = .28 |
|  | ExG-based | 4.5 [2.5 – 11.0] | 7.0 [3.0 – 12.5] | *U* = 3.16*e*^4^, *p* = .02,  *r* = .11, **adj-*p*_(3)_ = .08*** |
|  | HRVm-based | 2.0 [.5 – 6.5] | 3.75 [1.0 - 10.5] | *U* = 3.50*e*^4^, *p* = .003,  *r* = .15, **adj-*p*_(1)_ = .03*** |

**Table S3.** Table showing differences in bout durations between the RBD and OSA for each sleep stage and for each scoring method (manual, ExG-, and HRVm-based). Median and interquartile ranges (IQR) were reported for both populations. Asterisks indicate when population differences were significant after multiple testing correction.

| **Sleep Stage** | **Disorder** | **Scoring Method** | **Bout Duration Median [IQR]** | **Statistical Comparison: Kruskal-Wallis *H*-statistic, *p*-value, eta squared effect size** | **Post-hoc Comparison** | **Post-hoc statistics: Mann-Whitney *U-*statistic, *p*-value, rank-biserial effect size, adjusted *p*-value** |
| --- | --- | --- | --- | --- | --- | --- |
| Wake | RBD | Manual | .5 [.5 - 2.0] | *H*(2) = 11.93,  *p* = .003, η^2^ = .003 | Manual vs. ExG | *U* = 7.68*e*^5^, *p* = .01,  *r* = .05, **adj-*p*_(17)_ = .01*** |
|  |  | ExG-based | 1.0 [.5 - 2.5] |  | Manual vs. HRVm | *U* = 5.79*e*^5^, *p* = .001,  *r* = .07, **adj-*p*_(16)_ = .002*** |
|  |  | HRVm-based | 1.0 [.5 - 2.5] |  | ExG vs. HRVm | *U* = 5.30*e*^5^, *p* = .38,  *r* = .02, adj-*p*_(21)_ = .38 |
| Wake | OSA | Manual | .5 [.5 - 1.5] | *H*(2) = 1.49,  *p* = .48, η^2^ < .001 |  |  |
|  |  | ExG-based | .5 [.5 - 2.0] |  |  |  |
|  |  | HRVm-based | 1.0 [.5 - 2.0] |  |  |  |
| N1+N2 | RBD | Manual | 1.0 [.5 - 4.0] | *H*(2) = 249.35,  *p* < .001, η^2^ = .05 | Manual vs. ExG | *U* = 1.13*e^6^*, *p* < .001,  *r* = .26, **adj-*p*_(3)_ < .001*** |
|  |  | ExG-based | 3.0 [1 - 9.5] |  | Manual vs. HRVm | *U* = 7.86*e*^5^, *p* < .001,  *r* = .27, **adj-*p*_(4)_ < .001*** |
|  |  | HRVm-based | 3.5 [1 - 11.5] |  | ExG vs. HRVm | *U* = 9.79*e*^5^, *p* = .22,  *r* = .03, adj-*p*_(20)_ = .23 |
| N1+N2 | OSA | Manual | 1.5 [.5 - 4.5] | *H*(2) = 75.52,  *p* < .001, η^2^ = .06 | Manual vs. ExG | *U* = 1.13*e*^6^, *p* < .001,  *r* = .26, **adj-*p*_(2)_ < .001*** |
|  |  | ExG-based | 3.5 [1.0 - 10.0] |  | Manual vs. HRVm | *U* = 9.82*e*^5^, *p* < .001,  *r* = .29, **adj-*p*_(1)_ < .001*** |
|  |  | HRVm-based | 4.0 [1.0 - 12.5] |  | ExG vs. HRVm | *U* = 7.88*e*^5^, *p* = .03,  *r* = .05, **adj-*p*_(18)_ = .04*** |
| N3 | RBD | Manual | 1.5 [.5 - 5.0] | *H*(2) = 281.67,  *p* < .001, η^2^ = .08 | Manual vs. ExG | *U* = 5.65*e^4^*, *p* < .001,  *r* = .15, **adj-*p*_(15)_ < .001*** |
|  |  | ExG-based | 2.5 [.5 - 9.5] |  | Manual vs. HRVm | *U* = 3.04*e*^4^, *p* < .001,  *r* = .41, **adj-*p*_(8)_ < .001*** |
|  |  | HRVm-based | 6.5 [1.5 - 16.5] |  | ExG vs. HRVm | *U* = 2.05*e*^4^, *p* < .001,  *r* = .26, **adj-*p*_(12)_ < .001*** |
| N3 | OSA | Manual | 1.5 [.5 - 4.0] | *H*(2) = 100.69,  *p* < .001, η^2^ = .09 | Manual vs. ExG | *U* = 6.29*e*^4^, *p* < .001,  *r* = .23, **adj-*p*_(11)_ < .001*** |
|  |  | ExG-based | 3.0 [1.0 - 11.0] |  | Manual vs. HRVm | *U* = 4.16*e*^4^, *p* < .001,  *r* = .42, **adj-*p*_(6)_ < .001*** |
|  |  | HRVm-based | 5.5 [1.5 - 15.5] |  | ExG vs. HRVm | *U* = 2.64*e*^4^, *p* < .001,  *r* = .19, **adj-*p*_(14)_ < .001*** |
| REM | RBD | Manual | 2.0 [1.0 - 4.0] | *H*(2) = 150.26,  *p* < .001, η^2^ = .12 | Manual vs. ExG | *U* = 5.06*e*^4^, *p* < .001,  *r* = .40, **adj-*p*_(7)_ < .001*** |
|  |  | ExG-based | 4.5 [2.5 - 11.0] |  | Manual vs. HRVm | *U* = 8.07*e*^4^, *p* = .19,  *r* = .05, adj-*p*_(19)_ = .21 |
|  |  | HRVm-based | 2.0 [.5 - 6.5] |  | ExG vs. HRVm | *U* = 4.45*e*^4^, *p* < .001,  *r* = -.28, **adj-*p*_(10)_ < .001*** |
| REM | OSA | Manual | 1.5 [1.0 - 4.0] | *H*(2) = 83.19,  *p* < .001, η^2^ = .07 | Manual vs. ExG | *U* = 4.19*e*^4^, *p* < .001,  *r* = .51, **adj-*p*_(5)_ < .001*** |
|  |  | ExG-based | 7.0 [3.0 - 12.5] |  | Manual vs. HRVm | *U* = 7.28*e*^4^, *p* < .001,  *r* = .25, **adj-*p*_(9)_ < .001*** |
|  |  | HRVm-based | 3.75 [1.0 - 10.5] |  | ExG vs. HRVm | *U* = 5.11*e*^4^, *p* < .001,  *r* = -.22, **adj-*p*_(13)_ < .001*** |

**Table S4.** Table showing differences in bout durations between scoring methods (manual, ExG-, and HRVm-based) for each sleep stage and for both RBD and OSA. Median and interquartile ranges (IQR) were reported for each scoring method. When differences between scoring methods were significant, post-hoc comparisons were applied. Asterisks indicate when post-hoc comparisons were significant after multiple testing correction.

**REM Sleep Atonia Analysis**

The automated analysis of the REM atonia index (RAI) resulted in a RAI = .69 ± .21 for RBD subjects, where 66.7% of the RBD subjects showed RAI < .8 and 91.7% showed RAI < .9. In contrast, a limited number OSA subjects (RAI = .94 ± .06) reached RAI thresholds, with 5.6% showing a RAI < .8 and 13.9% showing a RAI < .9. For the RBD subjects, the RAI was correlated with the per-subject average performance and ambiguity scores, as visualized in Figure S6.

A significant correlation between the RAI and the ExG-based REM F1-score was observed, *r*(34) = .36, *p* = .03, indicating higher agreement between manual scoring and ExG-based ASSC when more atonia was detected during REM sleep. No significant relationship with the RAI was found for overall Kappa performance in ExG-based ASSC (*r*(34) = .19, *p* = .26), nor for the overall Kappa performance (*r*(34) = .04, *p* = .81) and REM F1-score (*r*(34) = .05, *p* = .764) using HRVm-based ASSC.

**
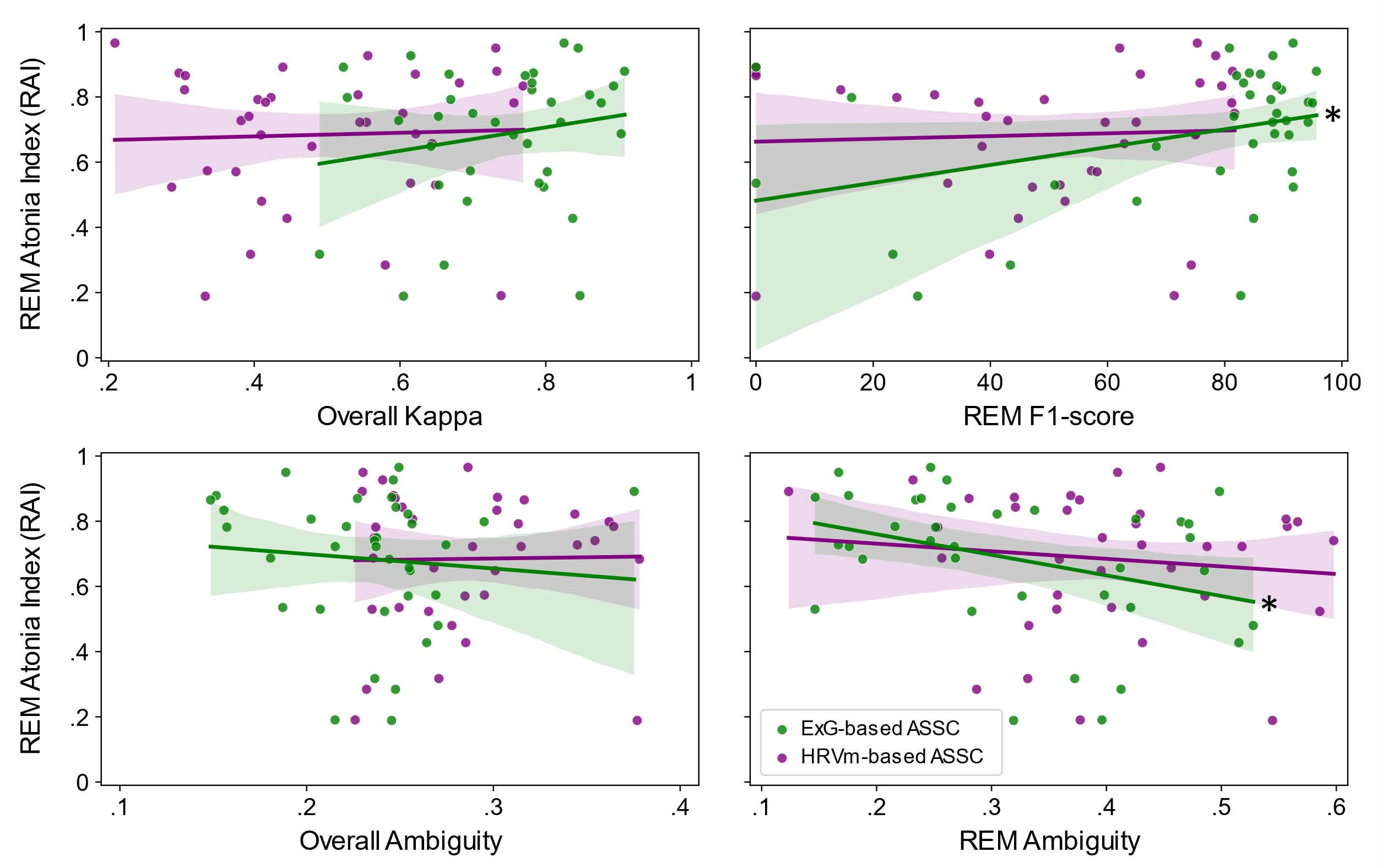
**Moreover, a significant correlation between the RAI and the ExG-based REM ambiguity was observed, *r*(34) = -.36, *p* = .03, indicating lower ambiguity in the ExG-based ASSC during REM classification when more atonia was detected. No significant relationship with the RAI was found for the overall per-subject ambiguity in ExG-based ASSC (*r*(34) = -.09, *p* = .586), nor for the overall per-subject ambiguity (*r*(34) = .02, p = .92) and REM-specific ambiguity (*r*(34) = -.12, *p* = .48) in HRVm-based ASSC.

**Figure S6**. Correlations between RAI and the overall Kappa (upper left), REM F1-score (upper right), overall ambiguity (lower left), and REM ambiguity (lower right) for both ExG-based (green) and HRVm-based (purple) ASSC in RBD subjects. Individual datapoints represent the per-subject averages. Solid lines represent predicted relation, with 95% confidence intervals in lighter shades. As indicated by the asterisks, for ExG-based ASSC, the correlations between the RAI and REM F1-score, as well as between the RAI and REM ambiguity are significant.

**Continuity and AHI**

Figure S6 shows the relationship between the AHI and the hypnodensity-derived continuity for both ExG-based ASSC and HRVm-based ASSC.


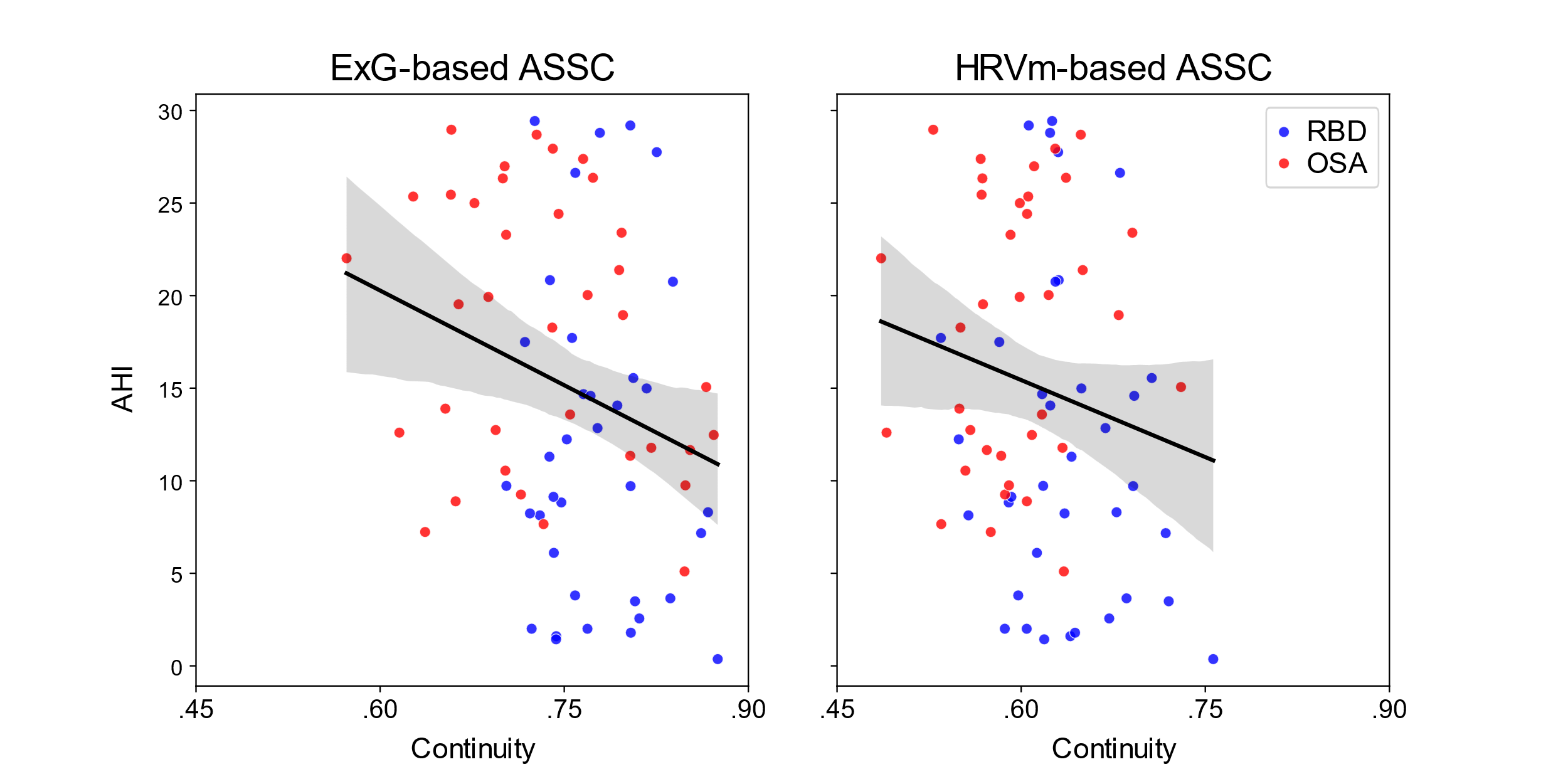


**Figure S6.** Correlations between the apnea-hypopnea index (AHI) and the per-subject average continuity derived from the ExG-based hypnodensity (left) and the HRVm-based Hypnodensity (right). Individual datapoints are shown for RBD (blue) and OSA (red), while the correlation is shown for both groups combined due to limited data. Solid black lines represent predicted relation, with 95% confidence intervals in grey. For ExG-based ASSC, a significant correlation was observed, *r*(70) = -.26, p = .03, indicating higher AHI is associated with lower continuity. No significant correlation with AHI was observed for the HRVm-based method, *r*(70) = -.17, *p* = .14.

**Isolated RBD and secondary RBD**

Table S7 and S8 show the statistical comparisons for agreement on the ASSCs and on hypnodensity-derived ambiguity and continuity.

|  | **Agreement for ExG-based ASSC** | | |  | **Agreement for HRVm-based ASSC** | | |
| --- | --- | --- | --- | --- | --- | --- | --- |
| **Stage** | **iRBD**  **(n = 20)** | **Secondary RBD**  **(n = 16)** | **Statistical Comparison** |  | **iRBD**  **(n = 20)** | **Secondary RBD**  **(n = 16)** | **Statistical Comparison** |
| Overall  (5 classes) | κ = .69 ± .08 | κ = .68 ± .12 | *T* = .48, *p* = .63,  *d* = .16 |  | - | - | *-* |
| Overall  (4 classes) | κ = .75 ± .10 | κ = .73 ± .13 | *T*= .56, *p* = .68,  adj-*p*_(5)_ = .92,  *d* = .19 |  | κ = .47 ± .16 | κ = .53 ± .15 | *T* = -1.21, *p* = .23,  adj-*p*_(3)_ = .78,  *d* = -.41 |
| Wake | F1 = 85.96 (13.35) | F1 = 85.94 (16.17) | *U* = 130, *p* = .35,  adj-*p*_(4)_ = .87,  *r* = -.96 |  | F1 = 65.05 (15.59) | F1 = 80.61 (15.68) | *U* = 82, *p* = .014,  adj-*p*_(1)_ = .14,  *r* = -2.48 |
| N1+N2 | F1 = 87.03 (4.52) | F1 = 87.36  (10.25) | *U* = 175, *p* = .64,  adj-*p*_(6)_ = .92,  *r* = .48 |  | F1 = 74.05 (16.42) | F1 = 73.69  (8.01) | *U* = 144, *p* = .62,  adj-*p*_(7)_ = .92,  *r* = -.51 |
| N3 | F1 = 81.36 (4.52) | F1 = 87.36 (10.25) | *U* = 203.5, *p* = .17,  adj-*p*_(2)_ = .78,  *r* = .21 |  | F1 = 54.75 (29.64) | F1 = 55.81 (46.81) | *U* = 168, *p* = .81,  adj-*p*_(9)_ = .94,  *r* = .25 |
| REM | F1 = 84.53 (12.85) | F1 = 87.13  (22.75) | *U* = 166.5, *p* = .85,  adj-*p*_(8)_ = .95,  *r* = .21 |  | F1 = 52.32 (32.38) | F1 = 58.42 (47.10) | *U* = 159, *p =* .99*,*  adj-*p*_(10)_ = .99,  *r* = -.03 |

**Table S7.** Sleep stage classification agreement in isolated RBD and secondary RBD between manual scoring and ExG-based ASSC (left), and between manual scoring and HRVm-based ASSC (right), for 5-class sleep staging (only for ExG-based ASSC), 4-class sleep staging, and per sleep stage for the 4-class classification. Overall classification performance indicates the mean and standard deviation per-subject Kappa agreement. Sleep stage-specific performance indicates the median per-subject F1-scores and interquartile ranges are shown. No significant differences between isolated RBD and secondary RBD were observed after multiple testing correction.

|  | **ExG-based ASSC** | | |  | **HRVm-based ASSC** | | |
| --- | --- | --- | --- | --- | --- | --- | --- |
| **Metric** | **iRBD**  **(n = 20)** | **Secondary RBD**  **(n = 16)** | **Statistical Comparison** |  | **iRBD**  **(n = 20)** | **Secondary RBD**  **(n = 16)** | **Statistical Comparison** |
| Overall  Continuity | .77 (.07) | .77 (.06) | *U* = 153, *p* = .84,  *r* = -.22 |  | .63 (.05) | .62 (.06) | *U* = 181, *p* = .51,  *r* = .67 |
| Overall  Ambiguity | .24 (.03) | .24 (0.07) | *U* = 147, *p* = .35,  adj-*p*_(8)_ = .43,  *r* = -.41 |  | .29 (.05) | .25 (.07) | *U* = 120, *p* = .10,  adj-*p*_(4)_ = .26,  *r* = -1.27 |
| Wake  Ambiguity | .20 (.11) | .16 (.10) | *U* = 100, *p* = .02,  adj-*p*_(2)_ = .15,  *r* = -1.91 |  | .25 (0.10) | .21 (.05) | *U* = 110, *p* = .06,  adj-*p*_(3)_ = .19,  *r* = -1.59 |
| N1+N2  Ambiguity | .20 (.05) | .24 (.05) | *U* = 87, *p* = .01,  adj-*p*_(1)_ = .11,  *r* = -2.32 |  | .27 (.09) | .27 (.07) | *U* = 157, *p* = .47,  adj-*p*_(10)_ = .47,  *r* = -.10 |
| N3  Ambiguity | .32 (.12) | .38 (.18) | *U* =142, *p* = .29,  adj-*p*_(6)_ = .41,  *r* = -.57 |  | .27 (0.1) | .29 (.10) | *U* = 130, *p* = .17,  adj-*p*_(5)_ = .35,  *r* = -.96 |
| REM  Ambiguity | .30 (.18) | .29 (.17) | *U* =157, *p* = .47,  adj-*p*_(9)_ = .47,  *r* = -.10 |  | .39 (.11) | .40 (.17) | *U* = 139, *p =* .26*,*  adj-*p*_(7)_ = .41,  *r* = -.67 |

**Table S8.** Statistical comparison between isolated RBD and secondary RBD for the hypnodensity-derived continuity (overall) and ambiguity (overall and sleep stage-specific) for both ExG-based ASSC (left) and HRVm-based ASSC (right). Medians and interquartile ranges are reported. For ambiguity, multiple testing correction was applied. No statistical differences were observed (after multiple testing correction) between isolated RBD and secondary RBD.

**Discussion on ASSC performance**

This study confirms earlier findings that agreement of ASSC models with manual scoring is generally worse in RBD than in other patient populations. Using a combination of EEG, EOG, and chin EMG, we obtained a κ = .74 and .69 in the RBD group for 4- and 5-stage classification respectively, and a significantly higher κ of .80 and .74 in the age- and sex-matched OSA group. Previous studies on sleep stage classification in RBD employing the same sensor combination have reported lower performances of κ = .48 and κ = .54 (Andreotti, et al., 2018; Cooray, et al., 2019). The superior performance in our study may be credited to the Somnolyzer ASSC system. Other studies that used ExG-based methods, including single-channel EEG, single-channel EOG, and combined EEG+EOG, have also reported decreased agreement for RBD when compared to healthy sleepers, insomnia, OSA, and heterogeneous sleep-disordered populations (Andreotti, et al., 2018; Cesari, et al., 2024; Cooray, et al., 2019; Cooray, et al., 2021; van der Aar, et al., 2024). In the present study, we specifically observed lower agreement in RBD for N1+N2 and REM stage classification. Decreased REM sensitivity is well-described in literature (Andreotti, et al., 2018; Cooray, et al., 2019; van der Aar, et al., 2024) and is, at least to some extent, attributed to REM sleep without atonia (RSWA), a distinct characteristic of RBD reflecting underlying pathophysiology (Boeve, et al., 2007; Sateia, 2014). The current study confirms these findings by reporting a positive relationship between the RAI and the agreement on REM classification. This can possibly be alleviated by targeted model training on datasets representative of RSWA characteristics, for example by training a model specifically on an RBD population (van der Aar, et al., 2024) or by fine-tuning a generally trained classifier to the characteristics of an RBD patient using earlier sleep recordings of the individual (Andreotti, et al., 2018). Both approaches yielded improved agreement, including for the detection of REM sleep.

Furthermore, we found a κ = .50 agreement in RBD between manual scoring and the 4-stage ASSC model utilizing HRV and body movements, which was significantly lower than the agreement in the matched OSA group (κ = .63). These results are in line with earlier findings, which documented lowest 4‑stage agreement in the presence of REM parasomnias - a group consisting of mainly RBD patients - when deriving HRV from either the same wrist-worn PPG sensor as in this study (κ = .55) (Wulterkens, et al., 2021) or from an ECG sensor (κ = .47) (Fonseca, et al., 2020). Specifically, for the RBD population, limited agreement on 3-stage classification (wake/non-REM/REM) has been reported using ECG (κ = .28) (Cooray, et al., 2021). The agreement difference can mainly be attributed to the large amount of heterogeneous data the currently used model was trained on. Moreover, the current method uses additional accelerometry and uses neural network layers instead of fully relying on a feature-based approach (Fonseca, et al., 2023). Interestingly, although the HRVm-based ASSC model targets different physiological processes to measure sleep, similar patterns in terms of sleep stage-specific performance were observed as with ExG-based ASSC. In both previous HRVm-based ASSC studies and the current study, the misclassification of REM sleep as N1+N2 was very pronounced. Although the highly complex and ‘black-box’ nature of the HRVm-based classifiers used in these studies prevents an interpretation of the ‘rules’ used to separate the two classes, we can outline a few hypotheses for possible reasons why this classification confusion occurs. Generally, REM is characterized by an alternating pattern of sympathetic and parasympathetic activity, with the former typically higher during REM than during non-REM, but lower than during Wake (Tobaldini, et al., 2013). It is possible that the presence of autonomic dysfunction as an early manifestation of α‑synucleinopathy in RBD blunts the sympathetic response we see in these patients, in turn making some HRV patterns resemble more those occurring during non-REM sleep. Second, the presence of RSWA might be also contributing to this misclassification. In the absence of RBD, periods of sympathetic activation during REM are accompanied by the characteristic muscle atonia. In contrast, during non-REM, sympathetic activations, e.g., due to arousals, are often accompanied by body movements (Tobaldini, et al., 2013). It is plausible that in RBD the simultaneous presence of sympathetic activity, and limb movements due to the absence of atonia is further confusing the classifier. Notably, the chin EMG-derived RAI did not correlate with agreement on REM classification for HRVm-based ASSC, suggesting a different expression of RSWA in the wrist-worn PPG and actigraphy acquired signals, but this should be further studied. Future work should explore whether models that use exclusively HRV, but not body movements, present similar misclassification patterns in this population. Additionally, it should be explored whether model adaptation techniques such as those described earlier for ExG-based ASSC also improve REM classification performance for HRVm-based approaches.

# References

Andreotti, F., Phan, H., Cooray, N., Lo, C., Hu, M. T., & De Vos, M. (2018). Multichannel sleep stage classification and transfer learning using convolutional neural networks. *40th annual international conference of the IEEE engineering in medicine and biology society (EMBC)*, 171-174.

Boeve, B. .., Silber, M. H., Saper, C. B., Ferman, T. J., Dickson, D. W., Parisi, J. E., . . . Tippman-Peikert, M. (2007). Pathophysiology of REM sleep behaviour disorder and relevance to neurodegenerative disease. *Brain*, 130(11), 2770-2788.

Cesari, M., Portscher, A., Stefani, A., Angerbauer, R., Ibrahim, A., Brandauer, E., . . . Rodriguez-Sanchez, A. (2024). Machine Learning Predicts Phenoconversion from Polysomnography in Isolated REM Sleep Behavior Disorder. *Brain Sciences*, 14(9), p.871.

Cooray, N., A. F., Lo, C., Symmonds, M., Hu, M. T., & De Vos, M. (2019). Detection of REM sleep behaviour disorder by automated polysomnography analysis. *Clinical Neurophysiology*, 130(4), 505-514.

Cooray, N., Andreotti, F., Lo, C., Symmonds, M., Hu, M., & De Vos, M. (2021). Proof of concept: Screening for REM sleep behaviour disorder with a minimal set of sensors. *Clinical Neurophysiology*, 132(4), pp.904-913.

Fonseca, P., Ross, M., Cerny, A., Anderer, P., van Meulen, F., Janssen, H., . . . Overeem, S. (2023). A computationally efficient algorithm for wearable sleep staging in clinical populations. *Scientific Reports*, 13(1), 9182.

Fonseca, P., van Gilst, M. M., Radha, M., Ross, M., Moreau, A., Cerny, A., . . . Overeem, S. (2020). Automatic sleep staging using heart rate variability, body movements, and recurrent neural networks in a sleep disordered population. *Sleep*, 43(9), zsaa048.

Sateia, M. J. (2014). International classification of sleep disorders. *Chest*, 146(5), 1387-1394.

Tobaldini, E., Nobili, L., Strada, S., Casali, K., Braghiroli, A., & Montona, N. (2013). Heart rate variability in normal and pathological sleep. *Frontiers in physiology*, 4, 294.

van der Aar, J. F., van den Ende, D. A., Fonseca, P., van Meulen, F. B., Overeem, S., van Gilst, M. M., & Peri, E. (2024). Deep transfer learning for automated single-lead EEG sleep staging with channel and population mismatches. *Frontiers in Physiology, 14*, 1287342.

Wulterkens, B. M., Fonseca, P., Hermans, L. W., Ross, M., Cerny, A., Anderer, P., . . . van Gilst, M. (2021). It is all in the wrist: wearable sleep staging in a clinical population versus reference polysomnography. *Nature and Science of Sleep*, 885-897.
